# Impact of pharmacist educational intervention on disease knowledge, rehabilitation and medication adherence, treatment-induced direct cost, health-related quality of life and satisfaction in patients with rheumatoid arthritis: study protocol for a randomized controlled trial

**DOI:** 10.1186/s13063-019-3540-z

**Published:** 2019-08-09

**Authors:** Atta Abbas Naqvi, Mohamed Azmi Hassali, Syed Baqir Shyum Naqvi, Muhammad Tariq Aftab

**Affiliations:** 1 Discipline of Social and Administrative Pharmacy, School of Pharmaceutical Sciences, Universiti Sains Malaysia, Minden, 11800 Penang, Malaysia; 2 Faculty of Pharmacy, Hamdard University, Madinat-ul-Hikmah, Karachi, 74400 Pakistan; 3 Department of Pharmacology, Islam Medical College, Sialkot, 51480 Pakistan

**Keywords:** Pharmacist intervention, Knowledge, Medication adherence, Rehabilitation, Physical therapy, Quality of life, Direct cost, Rheumatoid arthritis, Pakistan

## Abstract

**Background:**

The objective of this study is to evaluate the effectiveness of pharmacist intervention in improving disease knowledge, adherence to treatment, health-related quality of life (HRQoL) and direct cost of treatment. The study also documents patient satisfaction with pharmacist counselling as a quality control measure.

**Methods/design:**

This is a randomized, single-blind, two-arm, controlled trial in patients with rheumatoid arthritis visiting outpatient rheumatology clinics in Karachi, Pakistan. We will enroll patients with established diagnosis of rheumatoid arthritis over 3 months. The patients would be randomized through a computer-generated list into the control group, i.e., usual care or into the intervention group, i.e., pharmaceutical care, in a ratio of 1:1, after providing signed written consent. The study will take place in two patient-visits over the course of 3 months. Patients in the intervention group would receive intervention from the pharmacist while those in the control group will receive usual care. Primary outcomes include change in mean score from baseline (week 0) and at follow up (week 12) in disease knowledge, adherence to medications and rehabilitation/physical therapy. The secondary outcomes include change in the mean direct cost of treatment, HRQoL and patient satisfaction with pharmacist counselling.

**Discussion:**

This is a novel study that evaluates the role of the pharmacist in improving treatment outcomes in patients with rheumatoid arthritis. The results of this trial could set the foundation for future delivery of care for this patient population in Pakistan. The results of this trial would be published in a peer-reviewed journal.

**Trial registration:**

ClinicalTrials.gov, NCT03827148. Registered on February 2019.

**Electronic supplementary material:**

The online version of this article (10.1186/s13063-019-3540-z) contains supplementary material, which is available to authorized users.

## Background

Rheumatoid arthritis (RA) is a chronic inflammatory disease that mainly affects the joints and results in pain, swelling and decreased mobility. The disease leads to joint deformity and disability over time. The disease ranks third after osteoarthritis and gout as the major cause of disability and affects roughly 1% of the global population. Reduced mobility results in decreased productivity among patients and further worsens their quality of life [1]. While pharmacological treatment may be essential in managing the acute flares and episodic pain associated with the disease, self-care and home-based management of RA is another important area of care that patients need to incorporate to effectively manage RA.

Several studies have reported that self-care in RA effectively reduces acute flares [2, 3]. This could be done through patient education and counselling. Pharmacists provide pharmaceutical care that incorporates these areas of care. Pharmaceutical care is an individualized patient-centric health service delivered by pharmacists that incorporates, but is not limited to, disease education, therapy management, self-care and self-management of disease, therapy and motivational guidance.

Evidence from several randomized trials indicate that pharmacist-driven patient counselling, consultation, disease education and advice, as well as telephonic intervention, have improved patients’ self-care. Educating patients about managing RA empowers them in understanding signs and symptoms of disease and devising ways to reduce or limit aggravating factors. A randomized trial conducted by Petkova that involved a community, pharmacy-based, patient education program and demonstrated improved treatment outcomes in patients with RA [4]. Moreover, Mary and colleagues demonstrated a positive effect of a mobile phone short message service on medication adherence in patients with RA [5]. A systematic literature review by Leville and colleagues highlighted the role of the pharmacist as an adviser on issues related to medication management. The intervention performed by pharmacists has shown improvement in patients’ adherence to medication not only in rheumatological conditions but also in other chronic illnesses [6, 1]. Another randomized controlled trial (RCT) by Clifford and colleagues reported a significant drop in non-adherence to medication and in the incidence of medication-related problems after a 4-week follow up. This improvement was reported in patients with RA who had telephone intervention delivered by the pharmacist [7]. Moreover, Stockl and colleagues reported improved adherence to medication in patients with RA who had telephone consultations with the pharmacist [8].

Recent evidence that evaluates the pharmacist’s role in managing RA is scant. Moreover, to date there has been no study conducted in Pakistan to evaluate this issue. One has to look into the role and scope of pharmacy practice in Pakistan’s healthcare system to understand the need for such studies. Pharmacy services are still in the developmental phase and have not attained the level of healthcare delivery as in many economically developed and developing countries. Pharmacists are not involved in patient care to a great extent and are mostly associated with dispensing medicines. There are several barriers to the delivery of pharmaceutical care services in Pakistan [9]. The recognition of pharmacists as members of allied health teams who are involved in direct patient care is limited and is still debated. Studies have called for evaluation of the benefits of pharmacist inclusion in direct patient care [10]. Cardiovascular and endocrine illnesses are the leading cause of deaths in the Pakistani population. Studies have been conducted to report the impact of pharmacist-led care in patients with endocrine and cardiovascular illnesses [11–13].

Musculoskeletal illnesses are a major contributor to the individual’s decreased productivity, economic burden and mobility. Unlike other noncommunicable diseases, these illnesses may not result in death. However, they are the most common cause of persistent pain and impaired function [14]. They significantly decrease a person’s mobility, productivity and quality of life [15, 16]. The most common musculoskeletal illnesses are osteoarthritis, RA and osteoporosis [14, 16]. Data from the Institute of Health Metrics and Evaluation show that the prevalence of RA in Pakistan is 0.22% (0.22–0.25%). However, the figures for years lived with disability (YLDs) are high, i.e., 28.59 years (19.12–39.02), and for disease-adjusted life years (DALYs) were 39.64 years (28.84–51.75). These figures further rise to 0.92 (0.52–1.69) deaths due to RA, 40.12 (26.73–54.81) YLDs and 56.67 (40.22–75.92) DALYs in female patients with RA. All figures were reported out of 100,000 patients [17].

Since disease prevalence has increased in Pakistan of late and mainly affects middle-aged individuals, it is expected to affect their productivity, employability and income. This would worsen their health-related quality of life and adds to the economic burden of this disease on society. Therefore, there is a need to evaluate the impact of pharmaceutical-care-based educational intervention delivered by the pharmacist on treatment outcomes in Pakistani patients with RA.

### Objectives

The primary objective of the study is to evaluate the effectiveness of a multifactorial educational intervention on patient knowledge of RA and adherence to treatment. This intervention would be provided by a pharmacist. The secondary objectives are to assess the impact of the intervention on the cost of treatment, quality of life and patient satisfaction.

### Trial design

This trial is designed as a randomized, single-blind, parallel group trial. It has two arms, i.e., a control or “usual care” arm and an intervention or “pharmaceutical care” arm. Patients would be recruited and randomly assigned to either of the two arms using a computer-generated list and the patients’ medical record numbers. The allocation ratio would be 1:1. The trial has been designed as per the Standard Protocol Items: Recommendations for Interventional Trials (SPIRIT) and Template for Intervention, Description and Replication (TIDieR) checklists which are available as Additional files 1 and 2.

## Methods/design

### Design and setting

This study is a randomized, single-blind, parallel trial. The patients who participate in this study after providing signed consent would be randomly assigned to either the control group (CG), i.e., usual care, or intervention group (IG), i.e., pharmaceutical care. The allocation ratio will be 1:1. The participants in the IG would receive an educational intervention delivered by the pharmacist and provided with disease education literature. The participants in the CG would receive usual care without pharmacist intervention. The usual care in Pakistan’s healthcare system encompasses an appointment with a physician, which does not include pharmaceutical care. Usually, a pharmacist is limited to dispensing medicines to patients.

### Recruitment strategy

The patients will be recruited from the rheumatology departments at three tertiary care hospitals located in city of Karachi, Pakistan. Patients will be recruited through an advertisement displayed at the reception counters. The recruitment advertisement is still ongoing. A study officer would administer the consent form, which is available in both the English and Urdu languages, and enroll consenting patients in the study. The list of study sites is available as Additional file 3.

### Study plan

The process will start after patients attend the study explanation session and consent to participate. The patients who refuse to participate will be provided regular hospital care. Patients who consent to participate will sign the patient consent form and will be screened for eligibility. Ineligible patients will be excluded from the study and given regular hospital care. Eligible patients will be randomized by computer software into the IG or CG. Patients’ baseline data will be recorded. Enrolled patients’ demographic data will be documented at baseline. Patients will have their anthropometric measurements, i.e., weight, height, etc. noted in addition to their socio-demographic data. Moreover, the patients will be assessed for the outcome measures. The patients randomized into the IG will be provided pharmacist intervention. At the follow-up appointment, i.e., after 3 months (week 12) from baseline (week 0), the same data will be recorded from patients in both treatment groups.

**Fig. 1:**
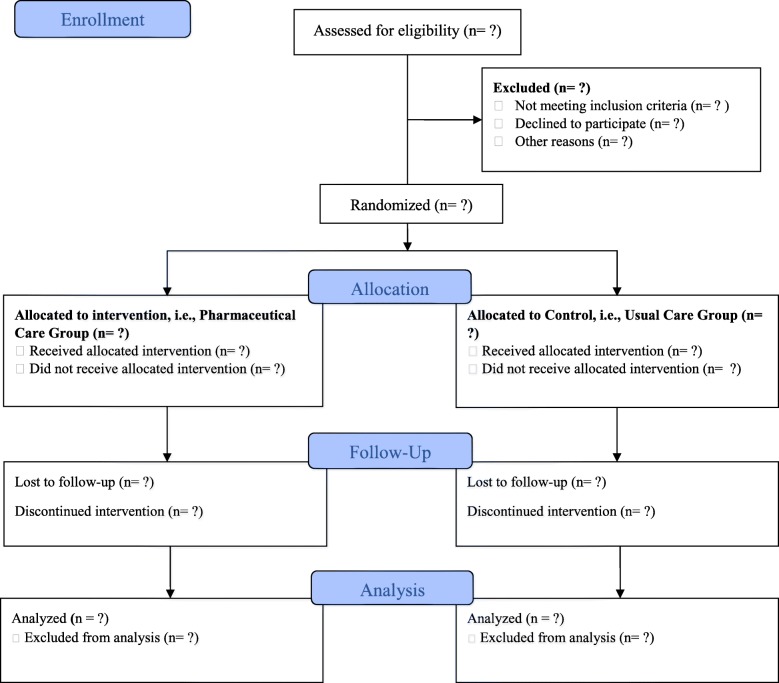
Study flow diagram

Attention is paid to achieving high patient-retention rates by comprehensively briefing the patients and answering their queries. In the case of loss to follow up, the rheumatologist and the hospital’s front desk staff will establish contact with the patient, try to convince them to participate and if not, document the reason for leaving the study. The whole study will encompass two visits in due course (Fig. 1).

### Participants

#### Inclusion criteria

The patients who meet the following criteria will be invited to participate in our study:

1. Patients suffering from RA diagnosed according to the 2010 American College of Rheumatology (ACR)/European League Against Rheumatism (EULAR) criteria
2. Patients diagnosed with RA based on the aforementioned criteria for at least 3 months prior to study invitation
3. Patients older than 18 years
4. Patients visiting outpatient clinics only

Patients attend a study explanation session and provide written consent to participate before enrolling in the study.

#### Exclusion criteria

Patients are excluded from the study based on the following conditions:

1. Musculoskeletal illnesses other than RA
2. Recent history of surgery or planned surgery for RA
3. More than three comorbidities
4. Abnormal laboratory measurements and receiving treatment for liver or kidney disease
5. Severe infection and/or completed a course of antibiotics in the last week
6. Advanced cardiovascular disease, severe allergies or a rare disease
7. Current participation or participation within the last 3 months in another clinical trial
8. Pregnancy, planning a pregnancy, breast feeding or other gynecological issues in women

### Randomization and allocation concealment

Patients will be randomly allocated to one of the two treatment groups using a randomization ratio of 1:1. The technique used will be simple randomization using a computer-generated list. The participants’ medical record numbers will be entered into the hospital’s software and a list of appointments will be generated with patients designated into the CG or the IG.

Allocation will be automatically concealed through the computer-generated list, which automatically allocates a clinic room number. For instance, if the computer enrolls a patient into the IG, it generates the appointment slip with a clinic room number in which a pharmacist will provide the intervention and vice versa. There are two room numbers, i.e., 1 = without pharmacist intervention and 2 = with pharmacist intervention. The patients and data collectors will be unaware of this number scheme. Patients in the CG will go to clinic room 1 and have their consultation with rheumatologist. The pharmacist will not provide the intervention. Before consultation, the patients will go to a consultation room where a data collector will wait with a data file printed on yellow-colored paper. If the computer enrolls a patient into the IG, the appointment slip will be printed with clinic room number 2. Patients in the IG will go to that clinic room where the rheumatologist and a pharmacist await them. The patients will undergo the intervention after consultation with the rheumatologist. Similarly, before the intervention, patients will enter a consultation room where the data collector will wait with a patient data file printed on white paper. The data collector and pharmacist will be unaware of the color scheme. Therefore, the pharmacist will be unaware of outcome assessment.

### Blinding

The investigators, data collectors, study observers, data entry operators and data analysis statistician will be blinded to the allocation. The patients will not be blinded to the pharmacists; however, they will be blinded to the intervention. The data collector will not know if the patient belongs to the IG or CG. The pharmacist will be blinded to the outcome assessment so that there will be less likelihood of intervention bias. The blinding scheme is shown in Table 1.

**Table 1:** Blinding scheme

Stake holders	Allocation scheme	Intervention allocation room	Pharmacist intervention	Data form color code	Outcome assessment	Data analysis
Study observers	A	NA	A	A	A	A
Study officer	NA	NA	NA	NA	NA	NA
Patients	NA	NA	NA	NA	NA	NA
Pharmacists	NA	NA	A	NA	NA	NA
Rheumatologists	NA	NA	A	NA	NA	NA
Data collectors	NA	NA	NA	NA	NA	NA
Data analysis technicians	NA	NA	NA	NA	NA	A

*A* Aware, *NA* Not aware

### Eligibility criteria for selection and training of pharmacists

An eligibility criterion has been outlined for pharmacists who would participate in the study. The minimum requirements for a pharmacist to be eligible to participate in the study and to be able to deliver the intervention were layed out. The applicant would be a graduate with Doctor of Pharmacy, i.e., Pharm.D degree obtained from an institute recognized by the Pharmacy Council of Pakistan [18]. The applicant should be registered as ‘Pharmacist’ in category, ‘A’, by the pharmacy regulatory body [19]. The registration certificate should be valid at the time of applying for participating in this study.There are two types for bachelor’s degrees awarded to pharmacists in Pakistan. The Bachelor of Pharmacy, i.e., B.Pharm and Doctor of Pharmacy, i.e., Pharm.D. The B.Pharm is a four-year degree while Pharm.D is relatively new degree that is completed over a course of five years with a residency component if the candidate wants to pursue a career in health sector. The Pharm.D degree has more clinically oriented courses and students learning is enhanced through clinical rounds [20]. Therefore, Pharm.D graduates would be included. The registration by regulatory body is important as it would be required in clinical practice. All applicants who lack in any of the abovementioned criteria would be excluded from study.The pharmacists involved in the study will be trained. A total of eight lectures of 1-h duration is planned over a span of 2 weeks to be given to participating pharmacists. These lectures will focus on selected topics on RA disease that are deemed important and helpful while educating patients. These will include disease epidemiology in Pakistan, symptoms and risk factors, brief information about diagnosis and treatment, the importance of physical therapy and rehabilitation, the importance of adherence to treatment and self-care techniques. Training will also include a lecture on the pharmacist’s role in self-care and home-based disease management and a lecture to reinforce patient counselling skills for pharmacists. The training will also encompass detailed briefing on the study protocol and administering the intervention. The training module and disease education literature will be provided to all pharmacists for the purpose of self-study at home. An assessment test based on the lectures will be conducted at the end. The training presentation is available as Additional files 4 and 5. Disease education literature for patients is available as Additional file 6.

### Intervention

The intervention consists of a pharmacist providing pharmaceutical care that will be in the form of an educational intervention. It will be delivered by the pharmacist as a single, face-to-face, structured session of 20–25 min duration. Moreover, specially designed RA disease education literature will be provided in both the Urdu and English languages for patients to use at home. An intervention delivery plan consisting of a checklist has been developed to assure uniformity of the intervention delivered to patients. The plan is available as Additional file 7.

**Fig. 2:**
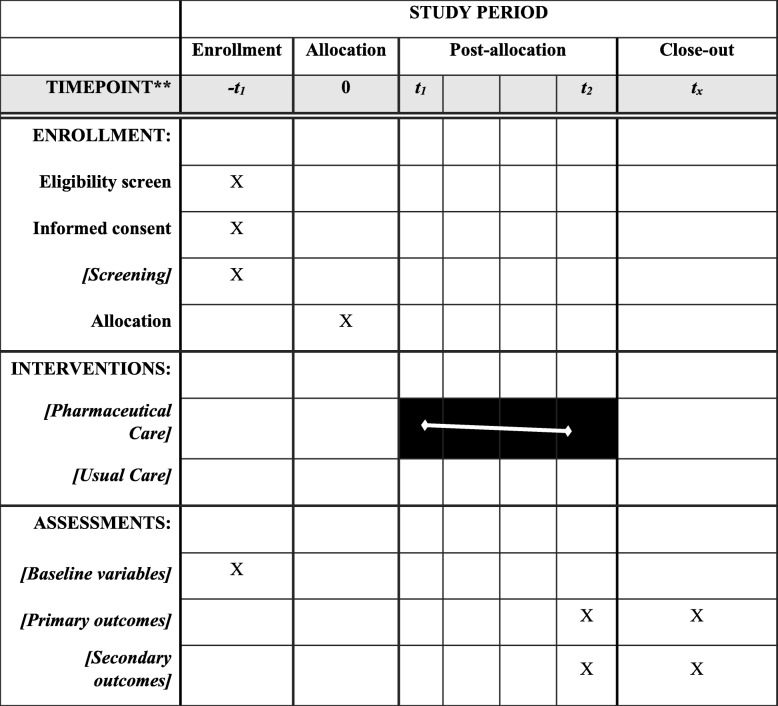
Schedule of enrollment, interventions and assessments

A specially designated counselling area in the rheumatology clinic serves as a venue for the intervention. The schedule of enrollment, intervention and assessment is provided in Fig. 2. The implementation will be observed by utilizing mixed models with random intercepts for pharmacists. The variance fraction explained will be observed by calculating the intraclass correlation coefficient using unconditional and conditional models [21].

### Outcomes

#### Primary outcome measurements

##### Treatment adherence

There are two modes of assessing treatment adherence, i.e., medication adherence and rehabilitation/physical therapy adherence. Both modes will be assessed after 3 months (week 12) from baseline (week 0). The Urdu version of the General Medication Adherence Scale (GMAS) [1] will be used for the assessment of adherence to medication and pharmacotherapy. The scale consists of eleven items that measure a patient’s adherence to medication and provide a score that could be interpreted as high, good, partial, low or poor adherence. There are four possible choices for each item, i.e., always, mostly, sometimes or never, which give a score of 0, 1, 2 or 3, respectively [22]. Adherence to rehabilitation/physical therapy will be assessed by utilizing the Urdu version of the General Rehabilitation Adherence Scale (GRAS). The scale consists of eight items that measure a patient’s adherence to physical therapy and provide a score that could be interpreted as high, good, partial, low or poor adherence. There are four possible choices for each item, i.e., always, mostly, sometimes or never, which give a score of 0, 1, 2 or 3, respectively (Naqvi AA: Development and validation of General Rehabilitation Adherence Scale (GRAS) in patients undergoing physical therapy for various musculoskeletal diseases. (unpublished work).

##### Knowledge of rheumatoid arthritis

Patient knowledge of RA will be assessed after 3 months (week 12) from baseline (week 0). The Rheumatoid Knowledge Assessment Scale (RAKAS) will be used to measure knowledge of RA disease. The RAKAS is a 13-item scale that contains questions related to knowledge, symptoms, treatment and risk factors. The items are multiple-choice questions (MCQs) and a correct answer awards a score while the wrong answers provide no score. A cumulative score is calculated, which is interpreted as excellent, good, low or poor knowledge (Naqvi AA: Development and validation of a novel Rheumatoid Arthritis Knowledge Assessment Scale (RAKAS) in Pakistani patients with rheumatoid arthritis. (unpublished work).

#### Secondary outcomes

##### Health-related quality of life (HRQoL)

The Urdu version of the 5-level European Quality of Life Scale (EQ-5D-5 L) will be used for assessing HRQoL. The EQ-5D-5 L is a generic tool to measure health and provides a numeric value for the patient’s health status. The scale comprises two parts, i.e., the descriptive health profile and the EQ visual analogue scale (VAS). The first component measures quality of life in the domains of mobility, self-care, usual activities, pain/discomfort and anxiety/depression. It measures patients’ responses on 5 different levels, i.e., having no problem, mild problem, moderate problem, severe problem and being unable/have extreme problem. The second component, EQ VAS, measures health status on a scale with two extreme points, i.e., best imaginable health and worst imaginable health. Patients are required to indicate their self-perceived health status on the scale. The score is then calculated as per the criteria specified by EuroQol [23, 24].

##### Direct cost of treatment

The mean direct cost of RA treatment after 3 months (week 12) from baseline (week 0) will be calculated in patients enrolled in the CG and in the IG. We consider direct cost of treatment as the cost incurred for appointments with rheumatologists, follow ups, medications and rehabilitation/physical therapy, aimed at minimizing any disease-related impairment [25, 26]. This will be documented using a specially designed data collection form.

##### Patient satisfaction

Patient satisfaction resulting from pharmacist-led pharmaceutical care will be assessed using a form for “Patient satisfaction feedback (PSF) on counselling” [27]. The proportion (percentage) of patients in the IG who were satisfied with the intervention after 3 months (week 12) from baseline (week 0) will be calculated.

### Feasibility assessments

In this trial we will have continuous assessment through designated health officials from hospital staff who would act as independent observers. These observers will be supervised by a designated member of the ethical review committee. Moreover, the committee will be updated separately by the officials and by the investigators on trial progress. This continuous assessment of trial progress will ensure three-tiered compliance of the study as per the approved protocol. The process will be assessed, i.e., the randomization process, blinding of data assessors, monitoring and management of data and the effectiveness, safety or any ethical issues pertaining to the intervention. The assessment of the study process will further help evaluate if the patients are properly briefed about the study and if the eligibility criteria need to be modified. Moreover, it will oversee the recruitment process as well as measures that are needed in case a patient withdraws his/her consent. Secondly, the assessment of study resources will encompass evaluation and/or modification in the numbers of materials used to document data that include stationery, computers, rooms, facilities, human resource and logistics. The data assessment will ensure proper data collection, data entry into the software and its validation. The assessment will also ensure blindness to allocation during data collection and analysis.

### Sample size

Few randomized controlled trials have evaluated the effect of pharmacists’ educational interventions on adherence and disease knowledge among patients with RA. We reviewed several studies that could serve as a reference for calculation of sample size based on treatment effect. A study conducted by Clifford and colleagues assessed the effect of pharmacist educational advice on improving adherence [7]. Another study by Stockl and colleagues was nonrandomized and was limited to evaluating the effects of a telephone intervention by either a pharmacist or a nurse, on patients’ adherence to injectable RA medicines [8]. Azzopardi and colleagues used a different outcome measure to ours and included patients with RA prescribed methotrexate only [28]. Similarly, in another RCT, Petkova measured a different outcome [3].

We considered a study by Clifford and colleagues as a standard and calculated our sample size based on the primary outcome, i.e., adherence [7, 29]. It was taken as a reduction in self-reported nonadherence from 16% to 9% considering a (two-side) alpha value of 0.05 and statistical power of 80%, i.e., beta = 0.2. Considering a drop-out rate of 20%, we would require a total of 619 patients to successfully demonstrate a positive and statistically significant effect.

### Data management

The data collection process will be conducted as per the defined protocol and standard operating procedures approved by the committee, to ensure genuine and good quality data. The patients will be given a study explanation session and after giving their consent, will be randomized and have their baseline data documented. After consultation, they will be informed about their follow-up visiting schedules. Patients with RA are usually given a follow-up appointment after 3 months. In addition, they are reminded about their visits via text reminders and followed by telephone calls if absent on the day of the appointment. The data will be collected by an independent assessor who will be blinded to the study objectives and intervention. After completion of the study, a data entry officer will enter the data into the software. This will be checked/validated by another person by comparison with the original patient data file. Any mistakes made during data entry will be rectified at this point.

### Statistical analysis

The statistical analysis will be conducted by a statistician who will be blinded to patient-group allocation. The analysis will be carried out using SPSS version 22 (IBM SPSS, Armonk, NY, USA). The analysis will consist of a per-protocol set that includes participants who have completed the study as per the defined protocol. The significance level will be set at 5% and a two-tailed test will be used. Continuous data will be descriptively analysed using the Mann-Whitney U test, while the chi-square test will be used to analyze categorical variables by comparing them in the two groups at the same time point. Confirmatory analysis will be carried out using the Wilcoxon signed-rank test to analyze differences in the mean values for disease knowledge, adherence to treatment and medications and the cost of treatment after 3 months, i.e., baseline within-subject analysis. Multinomial logistic regression will be used to interpret the odds ratios for the secondary outcome, i.e., patient satisfaction with pharmacist counselling. There will be no interim data analysis.

### Data monitoring

Data will be monitored in this study by independent health officials from the hospitals as designated by the ethical committee. The officials will monitor patient enrollment, study explanation sessions, patient consent documents, compliance to the defined protocol, data documentation and overall trial progress. A member of the ethical committee will serve as a protocol officer and will supervise those health officials. The protocol officer will serve as a communication link between the ethical committee and, health officials and investigators. The protocol officer, health officials and principle investigator constitute a steering committee that oversees the trial progress. In addition, the ethical committee will be updated by the steering committee on the progress of the study every 3 months.

### Data dissemination

The data forms collected from patients, would be handed over after statistical analysis, to the designated health officials from hospital staff who act as independent observers, and supervised by a designated member of the ethical review committee. The data forms will become property of the ethics committee and it will be at their discretion to decide if they wish to communicate the individual data with the rheumatologist and patients to appraise them about their treatment progress. This may prove helpful for patients and rheumatologists to analyze treatment progress and set future goals. Moreover, the results of the study will be communicated to the public in the form of publications. Authorship on publications would be decided as per the recommendations given by International Committee of Medical Journal Editors (ICMJE) [30]. The dataset obtained as a result of this study will be available solely for academic purposes on suitable request, subject to final approval from the ethics committee. The full study protocol is available at www.clinicaltrials.gov.

## Discussion

This study is designed as a randomized controlled trial that involves patients with RA. The nature of the disease demands self-care and home-based management; failure to understand or manage disease-aggravating symptoms lead to pain, mobility issues and increased suffering. This results in reduced productivity and quality of life [16, 31]. Therefore, it is important to derive ways to provide a patient-centric intervention that addresses these problems. These issues could be resolved through pharmaceutical care [12].

During the literature evaluation we came across the work of Abughosh and colleagues in which they evaluated the impact of telephone intervention by the pharmacist on medication adherence among patients with comorbid hypertension and diabetes mellitus [32]. The importance of patient-centered disease-education leaflets in increasing long-term patient knowledge has been demonstrated in a study [1]. Furthermore, in two randomized trials conducted in Pakistan, pharmaceutical care in the form of patient counselling significantly improved treatment outcomes in patients with hypertension. The outcomes included disease knowledge, medication adherence, HRQoL and blood pressure control [11, 33]. In this context, Lagger and colleagues reviewed 35 meta-analyses on the effectiveness of patient education, and concluded that patient education improves outcomes; however, the greatest benefits are seen when it is multi-dimensional and multi-disciplinary [2].

Therefore, we opted for a multi-dimensional intervention that could effectively address treatment outcomes in patients suffering from this disease. The composition of the intervention is based on previous research that has incorporated either of these aforementioned actions and evaluated their impact on improving patient-reported outcomes. The pharmaceutical care provided by a pharmacist in our study is multi-dimensional and encompasses patient counselling as well as disease education literature.

Outcome selection is an important area in designing clinical trials. The selected outcome must be relevant to patients, health care professionals and policy makers. Appropriate outcomes help in evaluating, disseminating and generalizing the findings of a trial [34]. There is a plethora of studies that evaluate disease knowledge, medication adherence and quality of life as prime treatment outcomes for chronic illnesses [4, 11, 32, 33]. However, treatment in RA may include rehabilitation/physical therapy to minimize mobility-related problems as a consequence of disease. It is worthwhile mentioning that rehabilitation/physical therapy was reported as an outcome by Kirkham and colleagues in their observational review of randomized trials over the last 50 years [34]. Therefore, adherence to rehabilitation/physical therapy is considered as another treatment outcome in our study. Thus, the primary outcomes are change in mean scores for disease knowledge, medication adherence and rehabilitation/physical therapy adherence. Since RA mostly affects middle-aged individuals, studies report that this age group is mostly affected with respect to productivity, which may add to the financial burden on the patient. Therefore, a change in the mean direct cost of treatment and patients’ HRQoL were considered as secondary outcomes.

One of the most important elements to assess in studies that evaluate the impact of counselling on treatment outcomes is the quality of counselling. Patient satisfaction could be considered as an indirect indicator of quality of service. Not only is it important from a subjective point of view, but this satisfaction could translate into future visits, trust and recommendation to others, thereby increasing the likelihood of pharmaceutical care delivery among the masses. Saleem and colleagues mentioned the absence of this indicator as a limitation in their study [11]. Therefore, patient satisfaction with pharmacist counselling is designated as another secondary outcome.

As of now, there have been no trials conducted in Pakistan that evaluate the effectiveness of a multi-dimensional pharmacist intervention in improving treatment outcomes in patients with RA. The results of this trial could set the foundation for future delivery of care for such patients in Pakistan and in other parts of the globe.

## Trial status

The trial commenced on 17 November 2018. The first patient was enrolled on 24 December 2018. The most recent version of the study protocol, i.e., USM-version 0.03, was approved by the ethical committee on 15 September 2018. The anticipated date of recruitment completion is 31 March 2019. The trial is active but not recruiting. Currently, the final results are being analyzed.

## Additional files

Additional file 1: SPIRIT 2013 checklist: recommended items to address in a clinical trial protocol and related documents. (DOC 122 kb)

Additional file 2: The Template for Intervention Description and Replication (TIDieR) checklist. (DOCX 30 kb)

Additional file 3: Study sites. (DOCX 13 kb)

Additional file 4: Rheumatoid arthritis refresher course and brief lecture on the pharmacist’s role and pharmaceutical care skills. (PDF 1607 kb)

Additional file 5: Study protocol and intervention training. (PDF 909 kb)

Additional file 6: Patient information booklet. (PDF 892 kb)

Additional file 7: Checklist for pharmacist intervention (to be completed by the rheumatologist). (DOCX 17 kb)

## Data Availability

The patient data pertaining to this study will be available to the study investigators and health officials.

## Abbreviations

ACR/EULAR: American College of Rheumatology/European League Against Rheumatism Collaborative Initiative
CG: Control group
GMAS: General Medication Adherence Scale
GRAS: General Rehabilitation Adherence Scale
HRQoL: Health-related quality of life
IG: Intervention group
MCQ: Multiple-choice question
RA: Rheumatoid arthritis
RAKAS: Rheumatoid Arthritis Knowledge Assessment Scale
SPSS: Statistical Package for Social Sciences
VAS: Visual analogue scale
