# Supplementary material for: Impact of pharmacist educational intervention on disease knowledge, rehabilitation and medication adherence, treatment-induced direct cost, health-related quality of life and satisfaction in patients with rheumatoid arthritis: study protocol for a randomized controlled trial
Source: Trials. 2019 Aug 9;20:488. doi: 10.1186/s13063-019-3540-z (PMC6688212; doi:10.1186/s13063-019-3540-z)
Supplement: Supplementary file 3 — Study sites. (DOCX 13 kb) [file 13063_2019_3540_MOESM3_ESM.docx]

**Study sites**

| S.No | Name | Address |
| --- | --- | --- |
| 1. | Site 1 | National Stadium Road,  Karachi, 74800, Pakistan |
| 2. | Site 2 | 111-A, 1/15, Ilyas Tower, Near Faisal Bank, Nazimabad No. 3.  Karachi, Pakistan. |
| 3. | Site 3 | Chand Bibi Road, Karachi-74200. Pakistan. |
| 4. | Site 4 | Block-B, North Nazimabad, Karachi-74700 |
| 5. | Site 5 | F-175, Block – 5, Clifton, Karachi-75600, Pakistan |
