# Supplementary material for: Impact of pharmacist educational intervention on disease knowledge, rehabilitation and medication adherence, treatment-induced direct cost, health-related quality of life and satisfaction in patients with rheumatoid arthritis: study protocol for a randomized controlled trial
Source: Trials. 2019 Aug 9;20:488. doi: 10.1186/s13063-019-3540-z (PMC6688212; doi:10.1186/s13063-019-3540-z)
Supplement: Supplementary file 4 — Rheumatoid arthritis refresher course and brief lecture on the pharmacist’s role and pharmaceutical care skills. (PDF 1607 kb) [file 13063_2019_3540_MOESM4_ESM.pdf]

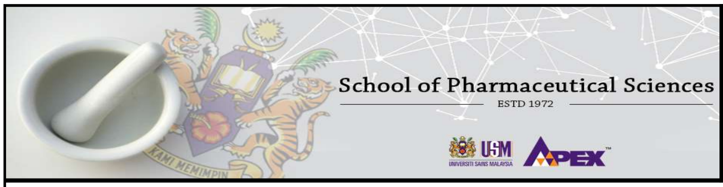

Rheumatoid arthritis refresher course and brief lecture on pharmacist's role and pharmaceutical care skills

For participating pharmacists

Universiti Sains Malaysia
Discipline of Social and Administrative Pharmacy
School of Pharmaceutical Sciences

Disclosure

- The lecturer has nothing to disclose.
- Permission was taken from all patients to use their pictures in preparation of this course.
- Permission was taken from hospitals and staff for use of photo capturing device in hospital vicinity.
- All information pertaining to patients is used strictly for academic purpose only.

Contents

- Rheumatoid arthritis refresher course
- Patient education
- Importance of medication adherence
- Importance of adherence to physical therapy in RA
- Role of a pharmacist
- Pharmaceutical care skills

Introduction

- Rheumatoid arthritis (RA) is a chronic systemic inflammatory disease of unknown cause.
- An external trigger (eg, cigarette smoking, infection, or trauma) that triggers an autoimmune reaction, leading to synovial hypertrophy and chronic joint inflammation along with the potential for extra-articular manifestations, is theorized to occur in genetically susceptible individuals.
- See the image.

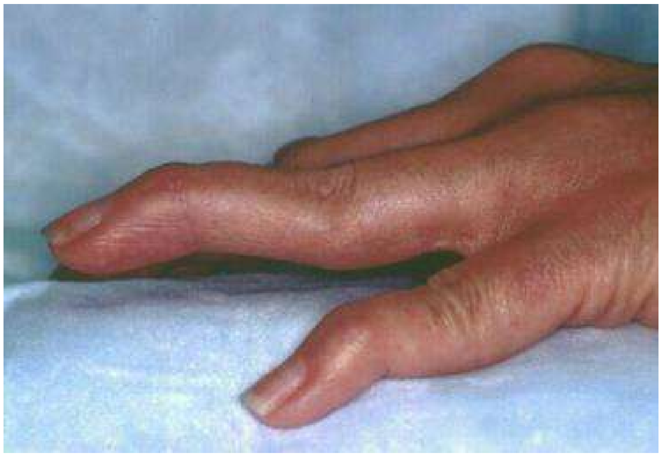

Signs and symptoms

- In most patients with RA, onset is insidious, often beginning with fever, malaise, arthralgias, and weakness before progressing to joint inflammation and swelling. Signs and symptoms of RA may include the following:

1. Persistent symmetric polyarthritis (synovitis) of hands and feet (hallmark feature)
2. Progressive articular deterioration
3. Extra-articular involvement
4. Difficulty performing activities of daily living (ADLs)
5. Constitutional symptoms
6. The physical examination should address the following:
7. Upper extremities (metacarpophalangeal joints, wrists, elbows, shoulders)
8. Lower extremities (ankles, feet, knees, hips)
9. Cervical spine

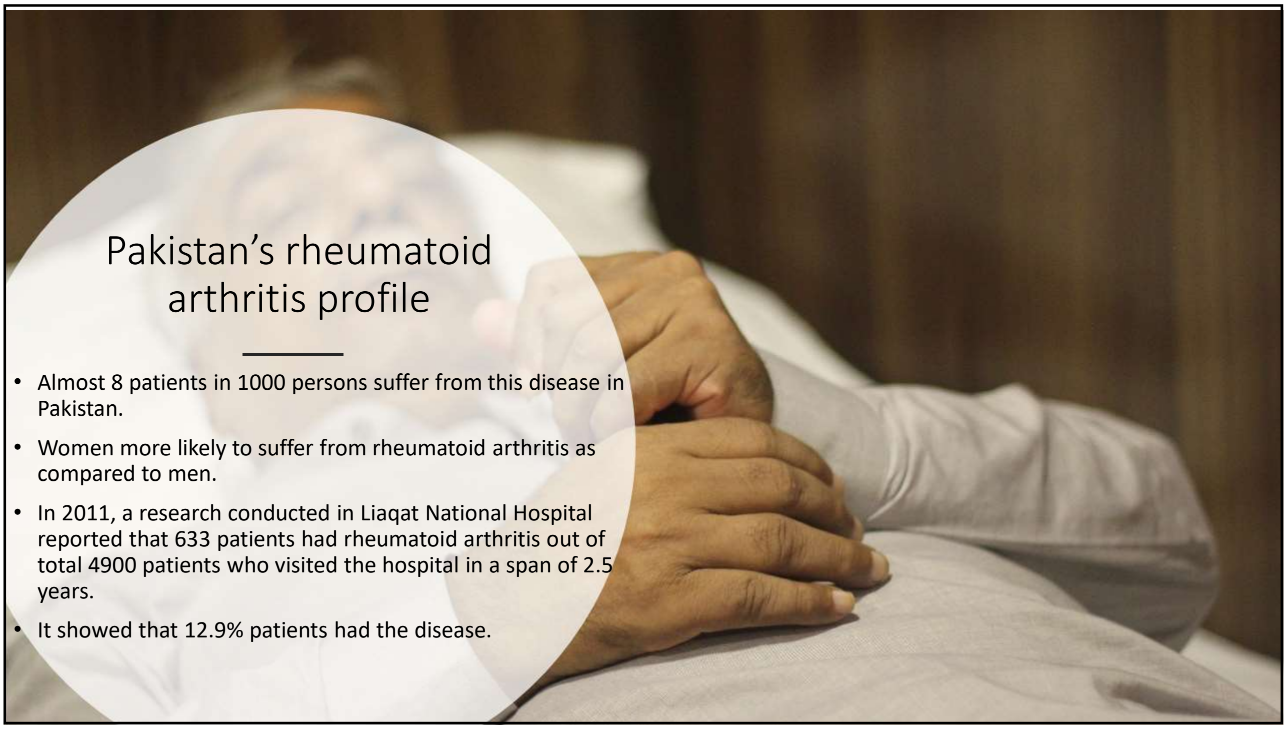

Pakistan's rheumatoid arthritis profile

- Almost 8 patients in 1000 persons suffer from this disease in Pakistan.
- Women more likely to suffer from rheumatoid arthritis as compared to men.
- In 2011, a research conducted in Liaqat National Hospital reported that 633 patients had rheumatoid arthritis out of total 4900 patients who visited the hospital in a span of 2.5 years.
- It showed that 12.9% patients had the disease.

Pakistan’s rheumatoid arthritis profile

- Data from the Institute of Health Metrics and Evaluation entails that the prevalence of RA in Pakistan is 0.22% (0.22% – 0.25%).
- However, the figures for years lived with disability (YLDs) were high, i.e., 28.59 years (19.12 – 39.02), and disease adjusted life years (DALYs) were 39.64 years (28.84 – 51.75).
- These figures further rose to 0.92 (0.52 – 1.69) deaths due to RA, 40.12 (26.73 – 54.81) YLDs and 56.67 (40.22 – 75.92) DALYs in case of female RA patients.
- All figures were reported out of 100,000 patients.

What you need to assess in physical examination?

- During the physical examination, it is important to assess the following:

1. Stiffness
2. Tenderness
3. Pain on motion
4. Swelling
5. Deformity
6. Limitation of motion
7. Extra-articular manifestations
8. Rheumatoid nodules

Diagnosis

- No test results are pathognomonic; instead, the diagnosis is made by using a combination of clinical, laboratory, and imaging features. Potentially useful laboratory studies in suspected RA include the following:

1. Erythrocyte sedimentation rate (ESR)
2. C-reactive protein level
3. Complete blood count
4. Rheumatoid factor assay
5. Antinuclear antibody assay

Diagnosis

Potentially useful imaging modalities include the following:

1. Radiography (first choice): Hands, wrists, knees, feet, elbows, shoulders, hips, cervical spine, and other joints as indicated
2. Magnetic resonance imaging: Primarily cervical spine
3. Ultrasonography of joints: Joints, as well as tendon sheaths, changes and degree of vascularization of the synovial membrane, and even erosions

- Joint aspiration and analysis of synovial fluid may be considered, including the following:

1. Gram stain
2. Cell count
3. Culture
4. Assessment of overall appearance

Treatment objectives

The aim of disease treatment is two pronged:

- Short term treatment includes reducing the pain, stiffness and inflammation of the joints
- Long term treatment goal includes preventing further joint damage, improve joint mobility and overall quality of life.

Management

Nonpharmacologic, nonsurgical therapies include the following:

- Heat and cold therapies
- Orthotics and splints
- Therapeutic exercise
- Occupational therapy
- Adaptive equipment
- Joint-protection education
- Energy-conservation education

Management

Nonbiologic disease-modifying antirheumatic drugs (DMARDS) include the following:

- Hydroxychloroquine
- Azathioprine
- Sulfasalazine
- Methotrexate
- Leflunomide
- Cyclosporine
- Gold salts
- D-penicillamine
- Minocycline

Management

Biologic tumor necrosis factor (TNF)–inhibiting DMARDs include the following:

- Etanercept
- Infliximab
- Adalimumab
- Certolizumab
- Golimumab

Management

Biologic non-TNF DMARDs include the following:

- Rituximab
- Anakinra
- Abatacept
- Tocilizumab
- Sarilumab
- Tofacitinib
- Baricitinb

Management

Other drugs used therapeutically include the following:

- Corticosteroids
- Nonsteroidal anti-inflammatory drugs (NSAIDs)
- Analgesics

Surgical treatments include the following:

- Synovectomy
- Tenosynovectomy
- Tendon realignment
- Reconstructive surgery or arthroplasty

Self care in rheumatoid arthritis

- Patients with or at risk of rheumatoid arthritis should eat a healthy diet that is rich in antioxidants.
- These antioxidants are helpful in reducing inflammation and factors responsible.
- The diet includes vegetables and fruits as well as fish. Other self care techniques are as follows:

1. **Body rest**

- Taking rest in between daily activities helps to maintain joint mobility.
- A small session of rest can reduce joint pain and inflammation.
- It is normally advised that taking a 10 minute rest after an activity helps in carrying out daily activities with ease.

Self care in rheumatoid arthritis

2. Physical activity

- Rheumatoid arthritis can result in reduce joint mobility.
- Reduced mobility may result in muscle weakness.
- This further makes it difficult for patients to carry out daily activity with the affected joint.
- To avoid this, a physical therapist can provide physical therapy including light exercises of the affected joint to strengthen the adjoining muscle.

Self care in rheumatoid arthritis

3. Topical therapy with hot and cold patches

- A hot and cold patch helps in reducing pain and inflammation.
- Hot patch helps in reducing joint stiffness whereas cold patch may reduce joint pain.

4. Use of dietary supplements

- Dietary supplements can also be taken to improve joint mobility, reduce stiffness and cartilage damage.
- One thing to bear in mind is to discuss with a doctor before using them as they may interfere with other medicines that you use.

Patient Education

Patient Education

- Patient education and counseling help to reduce pain, disability, and frequency of physician visits.
- These may represent the most cost-effective intervention for RA.

Informing patient of diagnosis

- Telling patients more than they are intellectually or psychologically prepared to handle (a common practice) risks making the experience so intense as to trigger withdrawal.
- Conversely, failing to address issues of importance to the patient compromises the development of trust.
- The patient needs to know that the primary physician understands the situation and is available for support, advice, and therapy as the need arises.
- The goal is to satisfy the patient's informational needs regarding the diagnosis, prognosis, and treatment in appropriate detail.

Patient Education

Discussing prognosis and treatment

- Patients and families do best when they know what to expect and can view the illness realistically.
- Many patients fear crippling consequences and dependency.
- Accordingly, it is valuable to provide a clear description of the most common disease manifestations.
- Without encouraging false hopes, the pharmacist can point out that spontaneous remissions can occur and most patients live independently without major disability.
- In addition, emphasize that much can be done to minimize discomfort and to preserve function.

Patient Education

Dealing with misconceptions

- Several common misconceptions regarding RA deserve attention. Explaining that no known controllable precipitants exist helps to eliminate much unnecessary guilt and self-recrimination.
- Dealing in an informative, evidence-based fashion with a patient who expresses interest in alternative and complementary forms of therapy can help limit expenditures on ineffective treatments.
- Another misconception is that a medication must be expensive to be helpful. Generic NSAIDs, low-dose prednisone, and the first-line DMARDs are quite inexpensive yet remarkably effective for relieving symptoms, a point that bears emphasizing.
- Active participation of the patient and family in the design and implementation of the therapeutic program helps boost morale and to ensure compliance, as does explaining the rationale for the therapies used.

Patient Education

- The family also plays an important part in striking the proper balance between dependence and independence.
- Household members should avoid overprotecting the patient and should work to sustain the patient's pride and ability to contribute to the family.
- Allowing the patient with RA to struggle with a task is sometimes constructive.

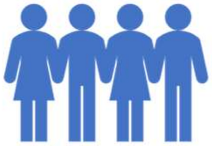

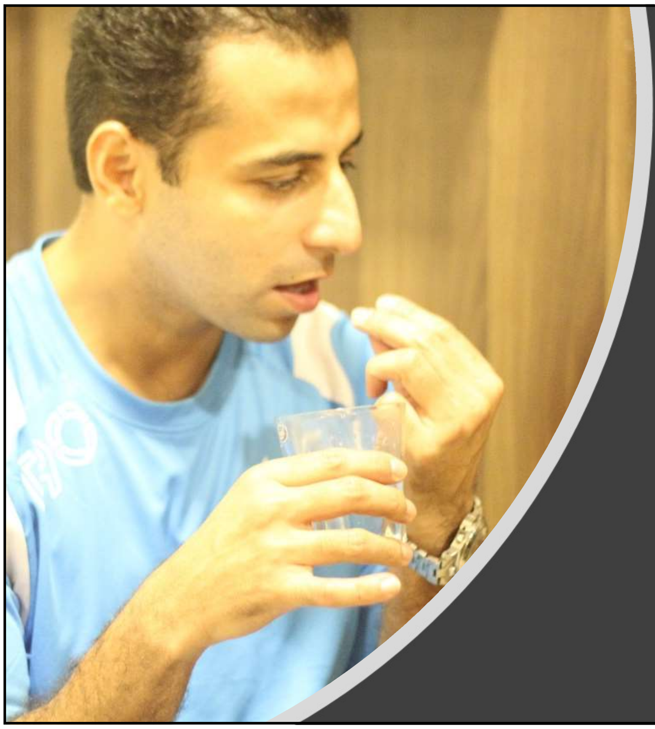

Medication Adherence

The Importance of Medication Adherence

What is adherence?

- Adherence is the amount of time someone is taking medications as instructed.

Adherence = Compliance + Persistence

Where,

1. Compliance is the medication consumption as instructed, % pills taken).
2. Persistence is the (duration of time during which medication is consumed, 1-discontinuation)

What is adherence?

- Adherence influenced by:

1. Extent
2. Duration
3. Severity of disease
4. Complexity of treatment regimen
5. Cost of medications
6. Education, social support, etc.

Factors and determinants of adherence

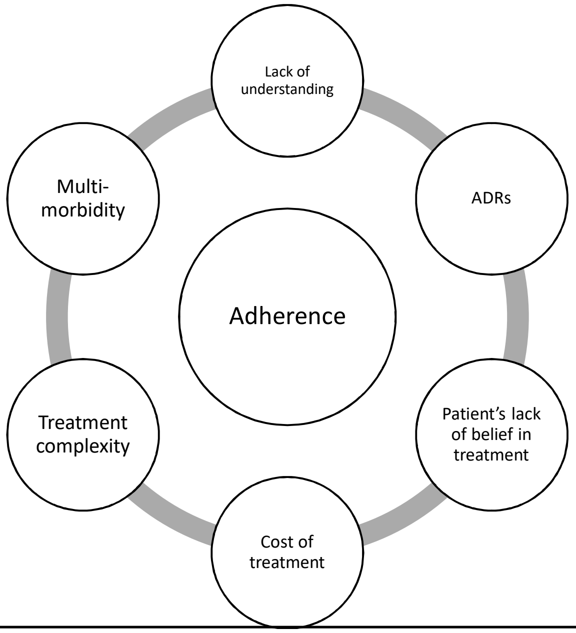

Medication adherence is best when…

- The diagnosis is short term/self-limiting
- The symptoms are predictable & improve with the medication
- The medication is taken for a short period of time
- The medication is taken once a day
- The medication does not have side effects
- The medication is inexpensive

Importance of adherence in RA

- Adhering to the prescribed therapy of rheumatoid arthritis is very important in achieving the treatment objectives.
- Research highlights that non-adherence to the medicines in this disease may result in rapid disease progression and cartilage damage which further leads to reduced mobility and deformity of the joints.
- Therefore, it is very important to strictly adhere to medicines to slow the disease progression and adequately adjust this disease in a patient's life.
- Patients forget their medicines during traveling or daily routine work.
- Patients may also forget to take the medicine during Eid, Ramadan and other festivities.
- Some patients may deliberately not take their medicines. This may be in response to feeling well, so the patient would think why should I take medicine when I feel fine without it? Else, if a patient suffers from a side effect of any medicine he/she would be prompted to discontinue the medicine thinking that the medicine given is wrong.
- Actually, both of these thoughts are wrong. It is important to educate patients that they must understand that they will be the sole recipient of benefit or disability as a result of medication adherence and non-adherence respectively.

Importance of adherence to physical therapy

- Physical therapy strengthens the joints and muscle.
- Rheumatoid arthritis resulted pain and stiffness can reduce the mobility of a patient.
- This causes muscles to become weak (because they are not used) thereby further making it difficult for a patient to carry out daily activities.
- Researchers agree that physical therapy may cause joint pain due to the exercises that a patient has to do.
- But on the other hand, such patients are physically more active, have more pain endurance and stamina as compared to a patient skipping physical therapy sessions.
- Hence, a physically active patient has more chance of living a quality life adjusted with disease as compared to a physically weak patient.

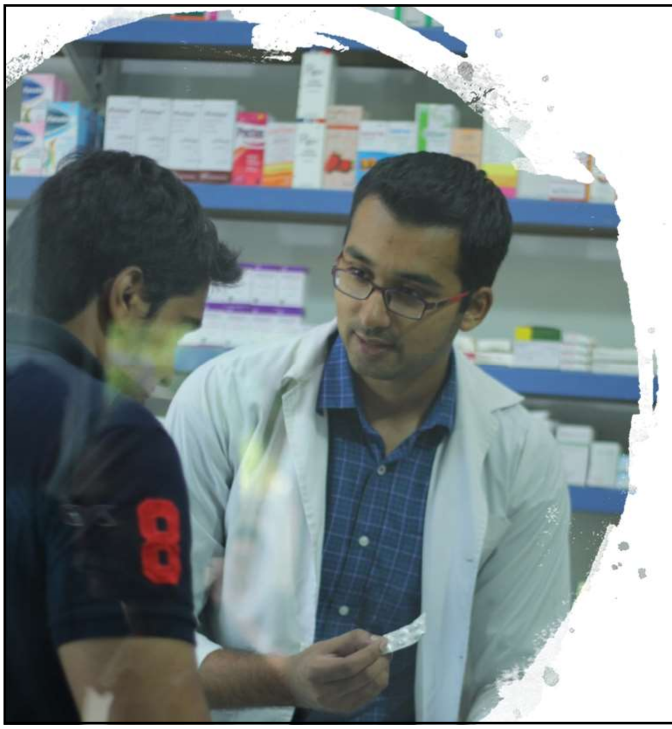

A brief introduction of pharmacists for patients

Who is a pharmacist and what is his/her role in patient care?

A brief introduction of pharmacist for patients

- Pharmacists and pharmaceutical care is a new concept in Pakistan, patients are not aware of its importance.
- Patients may have heard the term “pharmacist” in their daily life. But they know who is a pharmacist? Are they medical shop keepers? What does a pharmacist do? Why a pharmacist is present in the hospital? How a pharmacist is related to their disease management? These are few questions that come in a patient’s mind.
- Firstly, explain to patients that pharmacists are not medical shop keepers. They are equally qualified medical professionals as doctors are.
- One of the job functions of pharmacist is to prepare and dispense medicines to patients.
- Most patients may think about what exactly is the difference between a pharmacist and medical shop keeper? Both are doing the same job.

A brief introduction of pharmacist for patients

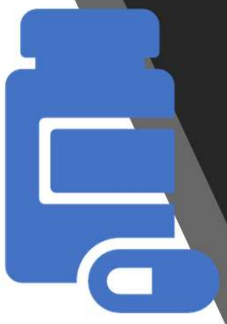

- It is important to explain the difference to patients. Some important points to clarify are:

1. A medical shop keeper dispenses medicine it but, a pharmacist not only dispenses the medicine, but may be helpful to educate patients about their disease.
2. Pharmacist can guide patients regarding management of disease condition it at home.
3. Pharmacist can give helpful advice in managing illness as pharmacists are drug information experts.
4. They can suggest dietary and lifestyle modifications which may help in minimizing adverse events related to disease conditions.

A brief introduction of pharmacist for patients

Sometimes, doctors are too busy in clinic and may not be able to give proper time to patients. They will listen to patients and write a prescription. In doing so, they will swiftly tell a patients how to take the medicines which they may not understand in the first place.

Instead of worrying, a patient can easily ask a pharmacist to interpret their prescription and guide them in treatment. Pharmacist can be a good alternative to doctors when it comes to disease education.

Also, a pharmacist also knows the alternate brands if a prescription medicine is not available in the pharmacy. He/she may be able to substitute a patient's medication requirements so that the treatment is not compromised.

A brief introduction of pharmacist for patients

Pharmacist may also be able to guide patients regarding how to take each medicine, which medicines in prescription needed to be taken in the morning or at night time. This will help patients manage their condition properly.

A pharmacist can also save money by suggesting cost effective alternatives available. For example, if the medicine is expensive, he may be able to suggest same medicine of a different company which is exactly the same but less expensive. So, a patient gets same but cost-effective treatment.

Some patients have more than 3 or 4 medicines. Sometimes due to an excessive number of medications, patient may have abdominal discomfort and adverse effects. A pharmacist can guide patients how to avoid those adverse effects.

Some medicines are sensitive to food, for example some medicines should not be taken with milk or on empty stomach. A pharmacist can tell patients correct way to take such medicines.

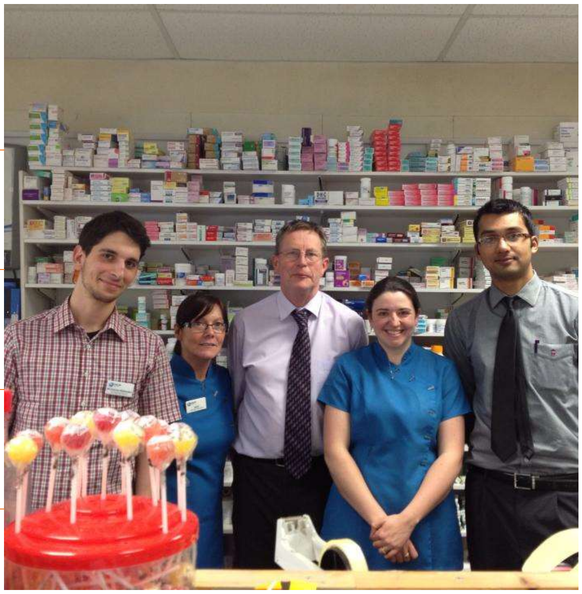

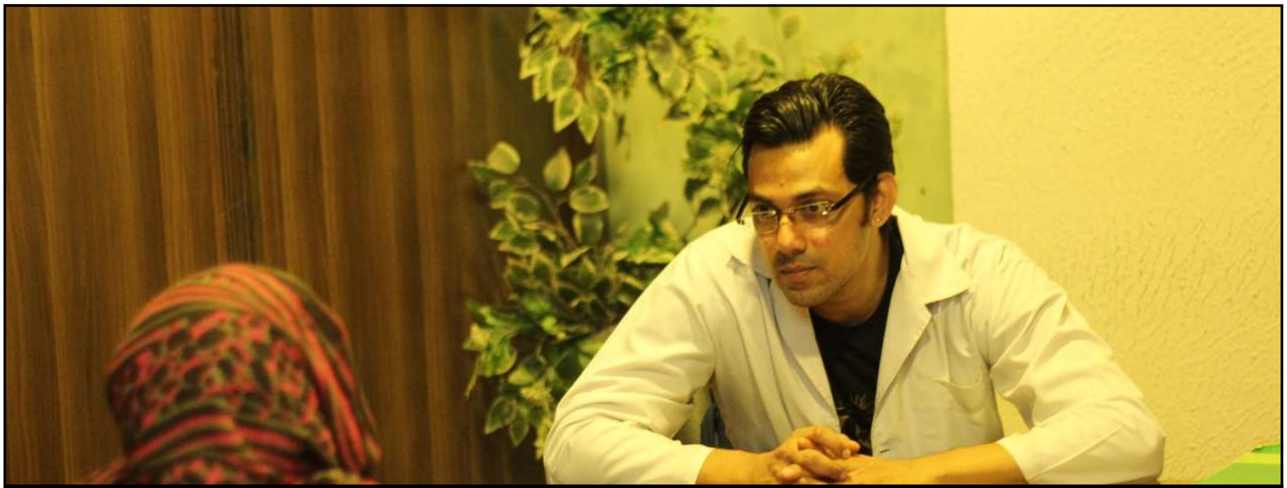

Pharmaceutical Care skills

Brief lecture for pharmaceutical care skills for practicing pharmacists

Pharmaceutical Care Skills

The practice of pharmaceutical care requires the mastery of a number of clinical skills.

The four sets of skills that are used on a continuous basis are:

1. Gathering information about your patient's medical conditions and drugs.
2. Evaluating and applying information to meet your patient's specific needs.
3. Communicating information to patients and professional colleagues.
4. Learning from your experience for the purpose of improving through reflective practice.

Pharmaceutical Care Skills

1. Obtaining Clinical Information from Patient

- The quality of the information you elicit will determine the quality of the care you can provide.
- This skill set integrates observational, interview, and physical assessment skills.

2. Observational Skills

- Observational skills require you to collect information with your eyes and your powers of deduction.

Patient variable	Examples of values to document
Age	•Approximate age in years or decades for older patients<br>•Example: A gentleman who appears to be in his 80's
Height, weight	Approximate height and weight<br>Example: Normal weight, slightly obese, very obese
Gender	Male, female
Overall health status	Assessment of physical/mental health<br>Example: Excellent, good, poor
Physical grooming and personal hygiene	Example: Neat, clean, unkempt, disheveled
Posture and general ability to ambulate	Level of physical activity<br>No difficulty ambulating<br>Difficulty ambulating<br>Example: good posture, poor posture
Ability to communicate	Language ability, use of hearing aid
Outward signs of illness	Normal or pallid skin tone<br>Energetic, lethargic<br>Example: Well nourished, malnourished

Patient variable	Examples of values to document
Apprehension, fear, agitation	Example: Anxious, distressed, preoccupied
Willingness and ability to participate	Cooperative, uncooperative<br>Example: Good historian, quiet

- Improving these observational skills first requires you to become conscious of the variables that will have an impact on your care plan.

Pharmaceutical Care Skills

3. Assessment skills

- The assessment interview is a purposeful conversation, which differs from casual or friend-to-friend conversations.
- Listen with an open mind
- Listen with empathy
- Becoming efficient at collecting patient information
- Collect only the information you will use
- Physical Assessment Skills
- Retrieving Information

4. Communication skills

- Communication with patients brings together many of the dimensions of pharmaceutical care practice, at the center of communication is the relationship between the patient and the practitioner.

Pharmaceutical Care Skills

Patient-Focused Communication:

- As a pharmaceutical care practitioner, you have **three primary objectives** to communicate with the patient:

1. To **elicit** necessary information from the patient to make your decisions.
2. To **negotiate** the terms of the goals of therapy and the patient's role in achieving them.
3. To **educate** the patient about the drug therapy she is receiving.

Pharmaceutical Care Skills

Patient-Focused Communication

- Educating the patient about drug therapy is most beneficial if you first understand the patient.

You will be most effective if you establish with the patient:

1. What your patient wants to know
2. What the patient already knows

- Your purpose for communicating is to fill the gap between what the patient wants to know and already knows in a way that a particular patient can best understand.

Pharmaceutical Care Skills

The best way to recognize this difference is to:

- determine the preferred language of the patient
- determine the level of comprehension best suited to the patient—this will determine the vocabulary/terms that are familiar to the patient
- identify any cultural or religious issues that are relevant to communicating with the patient.

Pharmaceutical Care Skills

You will want to explain the following to the patient:

- The reason for taking each medication (**Indication**).
- The specific **instructions** of how to take the medication explained in a manner the patient can understand.
- A description of how the patient will know that the medication is working well (**Effectiveness**).
- Explain the undesirable effects that might be expected (**Safety**).
- Be clear about what the patient should do if a dose of the medication is missed or if she takes an extra dose of the medication (**Adherence**).
- Inform the patient of when and how you intend to **follow-up** to evaluate effectiveness and safety of the medication.
- Educate patient to **contact** their physician if the medication is not working within the timeframe you discussed.

References

- [Guideline] Aletaha D, Neogi T, Silman AJ, et al. 2010 Rheumatoid arthritis classification criteria: an American College of Rheumatology/European League Against Rheumatism collaborative initiative. *Arthritis Rheum*. 2010 Sep. 62(9):2569-81. [Medline]. [Full Text].
- [Guideline] Anderson J, Caplan L, Yazdany J, et al, for the American College of Rheumatology. Rheumatoid arthritis disease activity measures: American College of Rheumatology Recommendations for use in clinical practice. *Arthritis Care Res (Hoboken)*. 2012. 64:640-7. [Medline]. [Full Text].
- [Guideline] Felson DT, Smolen JS, Wells G, Zhang B, van Tuyl LH, et al. American College of Rheumatology/European League Against Rheumatism provisional definition of remission in rheumatoid arthritis for clinical trials. *Arthritis Rheum*. 2011 Mar. 63 (3):573-86. [Medline]. [Full Text].
- [Guideline] Singh JA, Saag KG, et al. 2015 American College of Rheumatology Guideline for the Treatment of Rheumatoid Arthritis. http://dx.doi.org/10.1002/art.39480 (2015). *Arthritis Care and Research*. 2015. [Medline]. [Full Text].
- [Guideline] Smolen JS, Landewé R, Bijlsma J, et al. EULAR recommendations for the management of rheumatoid arthritis with synthetic and biological disease-modifying antirheumatic drugs: 2016 update. *Ann Rheum Dis*. 2017 Mar 6. 73(3):492-509. [Medline]. [Full Text].
- Donahue KE, Jonas DE, Hansen RA, Roubey R, Jonas B, Lux LJ, et al. Agency for Healthcare Research and Quality. Choosing Medications for Rheumatoid Arthritis. 2012 Apr. [Medline]. [Full Text].
- Kelly JC. Rheumatoid arthritis: Updated recommendations released. Medscape Medical News. Available at http://www.medscape.com/viewarticle/845495. May 28, 2015; Accessed: June 30, 2015.
- [Guideline] Smolen JS, Breedveld FC, Burmester GR, Bykerk V, Dougados M, et al. Treating rheumatoid arthritis to target: 2014 update of the recommendations of an international task force. *Ann Rheum Dis*. 2015 May 12. [Medline]. [Full Text].
- Carlens C, Hergens MP, Grunewald J, et al. Smoking, use of moist snuff, and risk of chronic inflammatory diseases. *Am J Respir Crit Care Med*. 2010 Jun 1. 181(11):1217-22. [Medline].
- Barton A, Worthington J. Genetic susceptibility to rheumatoid arthritis: an emerging picture. *Arthritis Rheum*. 2009 Oct 15. 61(10):1441-6. [Medline].

References

- Understanding arthritis. Arthritis Foundation. Available from: http://www.arthritis.org/about-arthritis/understanding-arthritis/
- Annelieke Pasma, Charlotte V. Schenk, Reinier Timman, Jan J. V. Busschbach, Bart J.F. van den Bemt, Esmeralda Molenaar, Willemijn H. van der Laan, Saskia Schrauwen, Adriaan van't Spijker, and Johanna M. W. Hazes. Non-adherence to disease-modifying antirheumatic drugs is associated with higher disease activity in early arthritis patients in the first year of the disease. Arthritis Res Ther. 2015; 17: 281.
- Ehtisham Akhter, Saira Bilal, Uzma Haque. Prevalence of arthritis in India and Pakistan: a review. Rheumatol Int (2011) 31:849–855.
- A. Farooqi and T. Gibson. Prevalence of the major rheumatic disorders in the adult population of north Pakistan. British Journal of Rheumatology 1998;37:491–49.
- Keep taking the pills – the critical importance of adherence in the management of rheumatoid arthritis. National Rheumatoid Arthritis Foundation (NRAS). Available from: http://www.nras.org.uk/keep-taking-the-pills-the-critical-importance-of-adherence-in-the-management-of-rheumatoid-arthritis
- Jet J. C. S. Veldhuijzen van Zanten, Peter C. Rouse, Elizabeth D. Hale, Nikos Ntoumanis, George S. Metsios, Joan L. Duda, and George D. Kitas. Perceived Barriers, Facilitators and Benefits for Regular Physical Activity and Exercise in Patients with Rheumatoid Arthritis: A Review of the Literature. Sports Med. 2015; 45(10): 1401–1412.
