# Supplementary material for: Impact of pharmacist educational intervention on disease knowledge, rehabilitation and medication adherence, treatment-induced direct cost, health-related quality of life and satisfaction in patients with rheumatoid arthritis: study protocol for a randomized controlled trial
Source: Trials. 2019 Aug 9;20:488. doi: 10.1186/s13063-019-3540-z (PMC6688212; doi:10.1186/s13063-019-3540-z)
Supplement: Supplementary file 5 — Study protocol and intervention training. (PDF 909 kb) [file 13063_2019_3540_MOESM5_ESM.pdf]

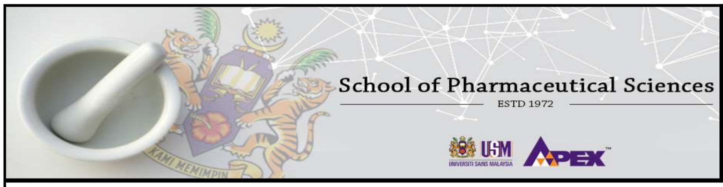

Study protocol and intervention training

For participating pharmacists

Universiti Sains Malaysia
Discipline of Social and Administrative Pharmacy
School of Pharmaceutical Sciences

Disclosure

- The lecturer has nothing to disclose.

Contents

- Study protocol
- Intervention training

Study design

Title of the study

Impact of pharmacist educational intervention on disease knowledge, rehabilitation and medication adherence, treatment induced direct cost, health-related quality of life and satisfaction in patients with rheumatoid arthritis: study protocol for a randomized controlled trial.

Objectives

The objectives of the study is to evaluate the effectiveness of a multifactorial educational intervention on patient knowledge regarding rheumatoid arthritis and adherence to treatment.

This intervention would be provided by a pharmacist.

The secondary objectives are to assess the impact of intervention on the cost of treatment, quality of life and patient satisfaction.

Background

- Rheumatoid arthritis is a chronic inflammatory disease that mainly affects the joints and results in pain, swelling and decreased mobility.
- The disease over the course of time, leads to joint deformity and disability.
- The disease ranks third as major cause of disability after osteoarthritis and gout and affects roughly 1% of global population.
- Decrease mobility in patients results in decreased productivity and further worsens their quality of life.
- While pharmacological treatment may be essential in managing the acute flares and episodic pain associated with the disease, self-care and home-based management of RA is another important area of care which patients need to incorporate to manage it effectively.

Background

- Several studies have reported that self-care in RA effectively reduces acute flares.
- This could be done through the use of patient education and counselling.
- Pharmacist provide pharmaceutical care that incorporates these areas of care.
- Pharmaceutical care is an individualized patient-centric health service delivered by pharmacists that incorporates, but is not limited to, disease education, therapy management, self-care and self-management of disease and therapy as well as motivational guidance.

Background

- Evidence from several randomized trials indicate that pharmacist-driven patient counselling, consultation, disease education and advice, as well as telephonic interventions, have improved self-care practices of patients.
- Educating patients about managing RA empowers them in understanding signs and symptoms of disease and devise ways to reduce or limit aggravating factors.
- Studies have reported the positive impact of education program on treatment outcomes of patients with rheumatoid arthritis such as adherence to medications.
- The role of pharmacist as adviser on issues related to medicines management has shown improvement in patients' adherence to medicines in not only rheumatological conditions but other chronic illnesses.
- Studies report a significant drop in non-adherence to medicines as well as incidence of medicines related problems after interventions delivered by pharmacists.

Problem statement

- There is a dearth of studies that evaluate pharmacists' role in managing RA in Pakistan.
- **Why?**

1. Pharmacy services is still evolving in Pakistan's healthcare system and has not reached the level of healthcare delivery as in many economically developed and developing countries.
2. Pharmacists are not involved in patient care to a greater extent and are mostly associated with dispensing of medicines.
3. The recognition of pharmacists as member of allied health team that is involved in direct patient care is limited and is still debated.

Study rationale

- Musculoskeletal illnesses such as osteoarthritis, rheumatoid arthritis and osteoporosis are a major contributor to an individual's decreased productivity, economic burden and mobility.
- They significantly decrease a person's mobility, productivity and quality of life.
- According to Institute of Health Metrics and Evaluation (IHME):

1. Prevalence of RA in Pakistan is 0.22% (0.22% – 0.25%).
2. Years lived with disability (YLDs) = 28.59 years (19.12 – 39.02).
3. Disease adjusted life years (DALYs) = 39.64 years (28.84 – 51.75).
4. In case of female patients; these figures further increase to 0.92 (0.52 – 1.69) deaths due to RA, 40.12 (26.73 – 54.81) YLDs and 56.67 (40.22 – 75.92) DALYs RA patients.
5. All figures were reported out of 100,000 patients.

Study rationale

- Since, the disease prevalence has increased in Pakistani population of late, and mainly affects the middle-aged individuals, it is expected to affect their productivity, employability and income.
- This would worsen their health-related quality of life and adds to economic burden of this disease on the society.
- Therefore, a need was felt to evaluate the impact of pharmacist-delivered pharmaceutical care on treatment outcomes in Pakistani patients with rheumatoid arthritis.

Study design

- **Design and setting**
- This study is a randomized single-blind parallel trial.
- The patients who participate in this study after signing of consent would be randomly assigned to either control group (CG), i.e., usual care or intervention group (IG), i.e., pharmaceutical care.
- The allocation ratio will be 1:1.
- The participants in the intervention group would receive an educational intervention by pharmacist and provided with a disease education literature.
- The participants in the control group would receive usual care without pharmacist intervention.
- **Recruitment strategy**
- The patients will be recruited from the rheumatology departments

Study design

- **Study plan**
- Patients attend study explanation session and consent to participate.
- Patients screened for eligibility and randomized into intervention and control groups, through software.
- The patients consulted and their baseline data is documented.
- The patients randomized into intervention group intervened by pharmacist.
- Patient randomized into control group receive usual care.
- Patients in both group assessed for the outcome measures after three months (week 12) from baseline (week 0).

Eligibility criteria for patients

Inclusion criteria

- RA patient diagnosed with rheumatoid arthritis according to the 2010 ACR/EULAR criteria.
- Patients diagnosed with RA based on above mentioned criteria for at least 3 months prior to invitation.
- Patients aged above 18 years
- Patients visiting out-patient clinics only.
- Before enrolling in the study, patients attended a study explanation session and provided a written consent to participate.

Eligibility criteria for patients

Exclusion criteria

- Patients with musculoskeletal illnesses other than rheumatoid arthritis
- Patients with a recent history of surgery or planned surgery for rheumatoid arthritis
- Patients with more than three co-morbidities
- Patients with any lab abnormalities, being treated for liver or kidney disease, had a severe infection or have completed antibiotic course in the past week.
- Patients with advanced cardiovascular disease, severe allergies or a rare disease.
- Patients who are currently participating in other clinical trial or have participated in past three months.
- Pregnant patients, planning to become pregnant, breast feeding and females with other gynecological issues were not included.

Intervention and usual care

Intervention

- A pharmacist will provide pharmaceutical care that will be in the form of an educational intervention based on refresher course and checklist.
- It will be delivered as a single, face-to-face, structured, session of 20 – 25 minutes duration.
- Specially designed rheumatoid arthritis disease education literature will be provided in both Urdu and English languages to patients for use at home.

Usual care

- Patients in usual care will have their appointment with rheumatologist that would not include pharmaceutical care.

Study design

Outcome measures:

1. Primary:

- *Adherence*

- General Medication Adherence Scale (GMAS) Urdu version for medication adherence
- General Rehabilitation Adherence Scale (GRAS) Urdu version for physical therapy adherence

- *Disease knowledge*

- Rheumatoid Arthritis Knowledge Assessment Scale (RAKAS) Urdu version

2. Secondary

- *Cost of treatment*

- A data collection form

- *Health-related quality of life (HRQoL)*

- The Urdu version of the five-level European Quality of Life Scale (EQ-5D-5L)

- *Satisfaction with intervention*

- Patient Satisfaction Feedback form

	STUDY PERIOD						
	Enrolment	Allocation	Post-allocation				Close-out
TIMEPOINT**	$-t_1$	0	t_1			t_2	t_x
ENROLMENT:							
Eligibility screen	X						
Informed consent	X						
[Screening]	X						
Allocation		X					
INTERVENTIONS:							
[Pharmaceutical Care]			←	—	→		
[Usual Care]							
ASSESSMENTS:							
[Baseline variables]	X						
[Primary outcomes]						X	X
[Secondary outcomes]						X	X

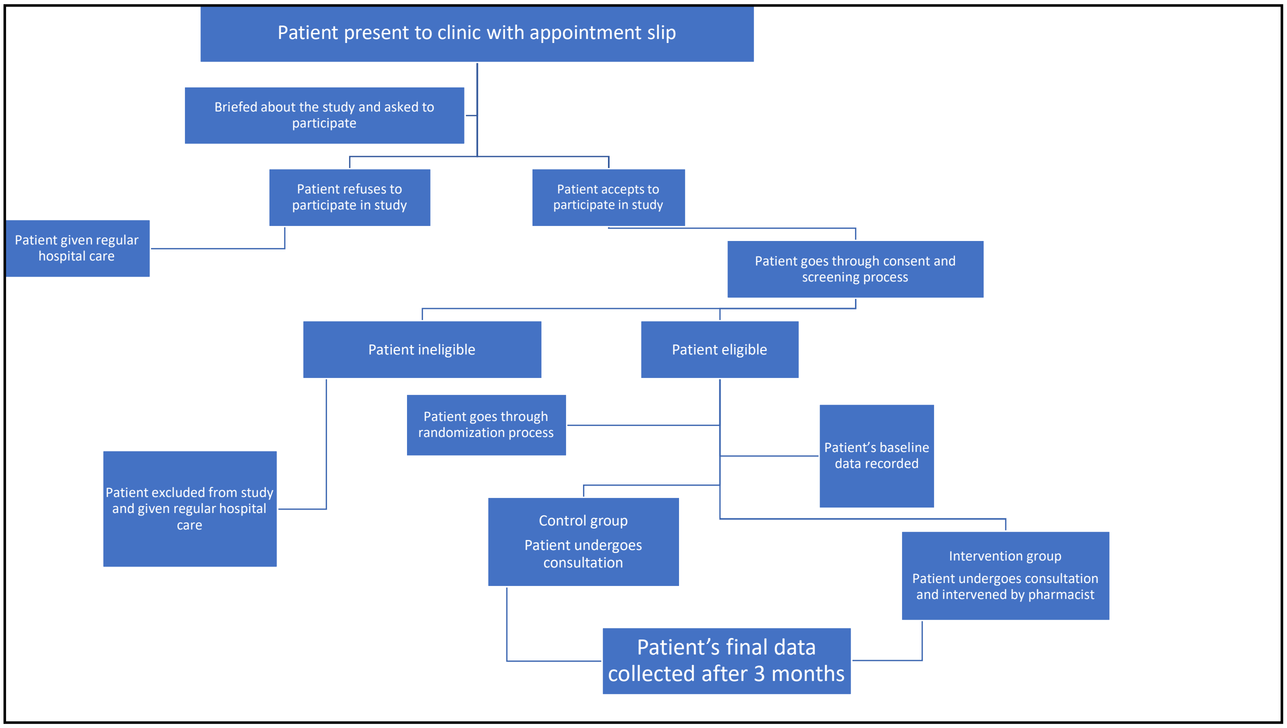

Process description

- Patients eligible for study will go through randomization process and will have their baseline data recorded.
- Patients will undergo consultation with rheumatologist and intervened by pharmacist (if in intervention group) or usual care, i.e., no intervention (if in control group).
- Patients will be followed up at week 12 from baseline (week 0) for their final data collection.
- Final data collection process will be same as baseline and would use the same tools.

Forms and materials

1. Consent form
2. Patient information questionnaire
3. GMAS
4. GRAS
5. RAKAS
6. EQ-5D-5L
7. Data collection form for cost of treatment
8. Patient satisfaction Feedback form (PSF) on couseling
9. Patient education booklet

Intervention overview

The intervention would consist of the following:

1. **Brief introduction of pharmacist**

- You would give an introduction to pharmacists and their role in patient care.
- You will be also explain the difference between pharmacist and medical shop keepers and highlight when and how pharmacist can help patients.

2. Patient education

- A pharmacist would be required to brief patients about the rheumatoid arthritis disease.

3. Explain importance of adherence to treatment

- A pharmacist need to explain the importance of adhering to medications and physical therapy.

Intervention overview

4. Medication review

- Review patient medication and give advice on managing drug therapy.
- Educate patients about proper dosage schedule, frequency and quantities of medicines to be taken at appropriate timings.
- Suggest cheaper drug alternatives to decrease the cost if patient and rheumatologist agrees.

5. Quality of life

- Suggest home based self-care techniques and approaches based on patient’s circumstances that could help in increasing productivity and mobility.

Note

- The intervention must be provided based on the training given.
- Pharmacists are required to remain in defined scope of disease education as per the refresher course.
- Pharmacist should make use of pharmaceutical care skills taught during training sessions while counseling the patients.
- At the end of the counseling session, the pharmacist would provide the rheumatoid arthritis disease education booklet to patients for use at home.

Checklist for pharmacist intervention

(to be completed by rheumatologist)*

I can confirm that the pharmacist provided the following to my patient:

- ❑ greeted the patient and made him comfortable
- ❑ asked the patient about his/her present complains
- ❑ provided a brief educational session about rheumatoid arthritis disease to patient
- ❑ explained the importance of adhering to medications prescribed
- ❑ explained the importance to physical therapy adherence
- ❑ reviewed medications, explained the dosage regimen and educated patients regarding dosage schedule
- ❑ suggested generic alternatives to reduce the cost of treatment (if applicable)*
- ❑ suggested home-based self care techniques and approaches aimed at improving productivity and mobility

I can further confirm that:

- ❑ the whole session was conducted in Urdu language
- ❑ the patient was able to understand the instructions given by pharmacist
- ❑ patient was able to follow the lecture pace
- ❑ the pharmacist exhibited professionalism while executing intervention
- ❑ disease education booklet was provided to patients
