# Supplementary material for: Impact of pharmacist educational intervention on disease knowledge, rehabilitation and medication adherence, treatment-induced direct cost, health-related quality of life and satisfaction in patients with rheumatoid arthritis: study protocol for a randomized controlled trial
Source: Trials. 2019 Aug 9;20:488. doi: 10.1186/s13063-019-3540-z (PMC6688212; doi:10.1186/s13063-019-3540-z)
Supplement: Supplementary file 6 — Patient information booklet. (PDF 892 kb) [file 13063_2019_3540_MOESM6_ESM.pdf]

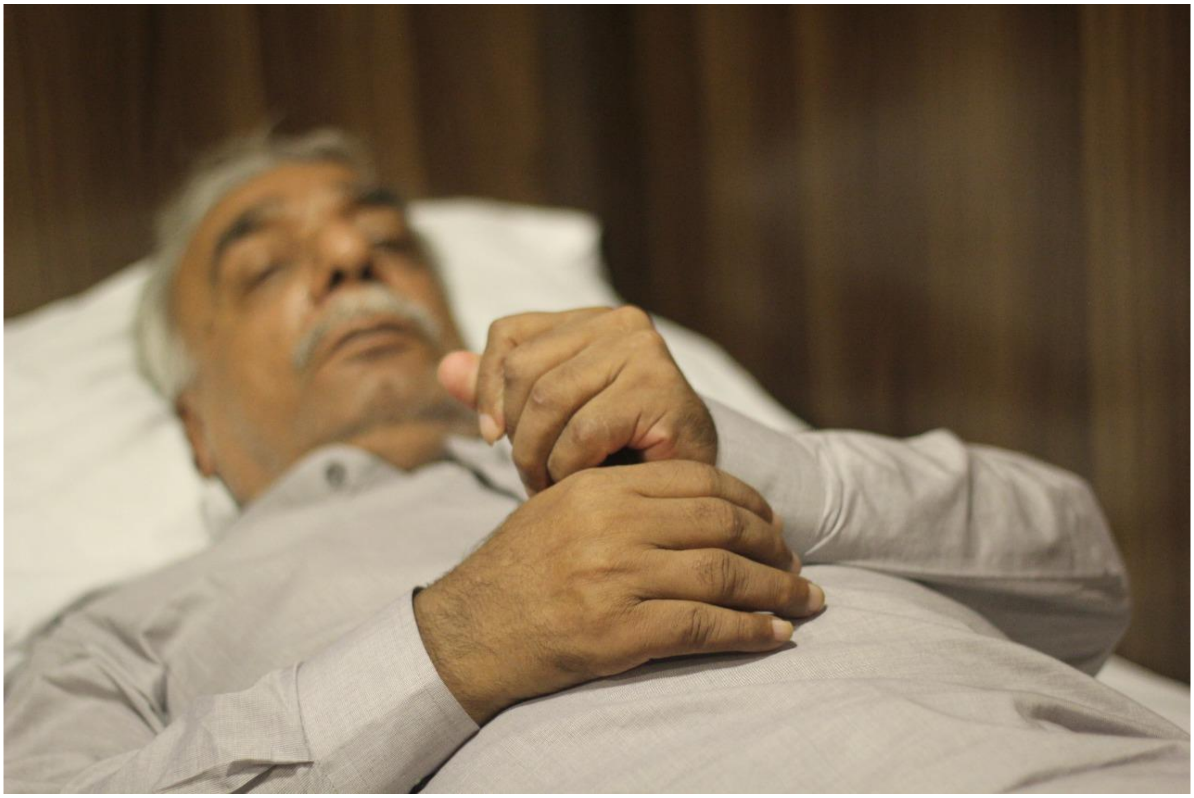

روماٹائڈ آرتھرائٹس کا علاج و احتیاط

مریض کے لئے معلوماتی کتابچہ

ڈاکٹر عطا عباس نقوی

ڈاکٹرمحمد اعضمی احمد حَسّالی

UNIVERSITI SAINS MALAYSIA

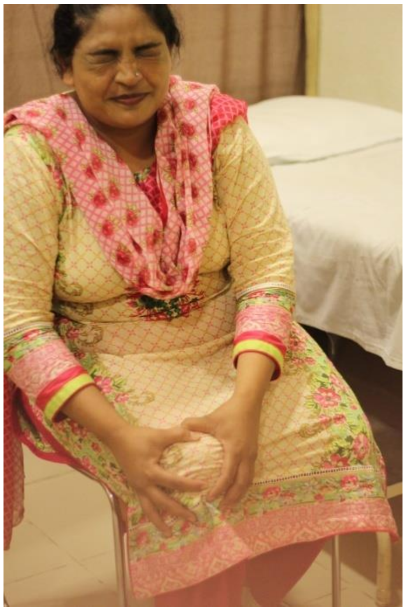

کیا آپ کو جوڑوں کا درد اور سوجن رہتی ہے؟ اور یہ درد روماٹائڈآرتھرائٹس کی وجہ سے ہے؟ روماٹائڈ آرتھرائٹس کیا ہے؟ یہ وہ مرض ہے جو ہمارے جوڑوں پر ہمارے معدافیتی نظام کے حملے کی وجہ سے پیدا ہوتا ہے۔ معدافیتی نظام ہمارے جسم میں موجود نظام ہے جو ہم کو مختلف قسم کے جراثیمی حملوں مثلاً سردی، بخار، حلق کے انفیکشن کے خلاف لڑنے کی صلاحیت دیتا ہے۔ جب یہ معدافیتی نظام ہمارے جوڑوں پرحملہ آور ہوجاتا ہے۔ ایسی صورت میں جوڑوں کا درد اور ورم پیدا ہوجاتا ہے۔ اگر اس درد اور سوجن کا علاج نہ کیا جائے اور اسے ایسے ہی چھوڑ دیا جائے تو اس سے جوڑوں کو نقصان پہنچتا ہے۔

ہمارے جوڑ نرم ہڈی پر مشتمل ہوتے ہیں جو جوڑوں کی ہڈیوں کے درمیان نرم گدّے کا کام کرتی ہیں۔ جوڑوں کو اس وقت نقصان پہنچتا ہے جب یہ نرم ہڈی مستقل درد اور ورم کا شکار رہتی ہے۔ اس کے نتیجے میں یہ نرم ہڈی سکڑ کر ایک فاصلہ پیدا کردیتی ہے اور جوڑ کی سخت ہڈی کو نقصان پہنچنے لگتا ہے۔

جب ایسا ہوتا ہے تو ہڈیاں آپس میں گِھسنے لگتی ہیں۔ درد اور سوجن میں اضافہ ہوجاتا ہے۔ اس کا نتیجہ یہ ہوتا ہے کہ جوڑوں کی حرکت کم ہوجاتی ہے اور ان میں نقص (بدصورتی) پیدا ہو جاتا ہے۔ سب سے اہم بات یہ ہے کہ ذہن میں رکھا جائے کہ یہ عمل آگے بڑھتا جاتا ہے اور بلآخر ناقابلِ تلافی ہوجاتا ہے۔ علاج پوری زندگی چلتا ہے۔ ان تمام نقصانات سے بچاؤ صرف اس صورت میں ممکن ہے کہ علاج فوری کیا جائے اور ایسی دوائیں استعمال کی جائیں جو جوڑوں کے نقصان کے عمل کو آہستہ کردیں۔ یہ دوائیں ڈیمارڈز (ڈی۔مارڈز) کہلاتی ہیں۔ روماٹائڈ آرتھرائٹس صرف گُھٹنے کے جوڑوں کو متا ثر نہیں کرتا بلکہ یہ کَلائی، ہاتھ، کوہنی اور ٹخنوں کے جوڑوں کو بھی متا ثر کرتا ہے۔

ہر بیماری کی کچھ علامات ہوتی ہیں لہذا کسی بیماری کی تشخیص کے لئے ان علامات کا جاننا ضروری ہوتا ہے۔ روماٹائڈ آرتھرائٹس کی علامات مندرجہ ذیل ہیں۔

۱۔ لمبے عرصے تک مثلاً 6 ہفتے یا اس سے زیادہ جوڑوں کا درد اور ورم کا موجود رہنا۔

۲۔ صبح جاگتے وقت جوڑوں میں سختی محسوس ہونا جو ۲۰ سے ۳۰ منٹ تک رہتی ہے۔

۳۔ ایک سے زائد جوڑوں کا متا ثر ہونا۔

۴۔ عام طور پر جسم کے دونوں جانب ایک ہی قسم کے جوڑوں کا متا ثر ہونا مثلاً اگر ایک گُھٹنا متاثر ہو تو دوسری جانب کے گُھٹنے پر بھی بیماری کا اثر ہوتا ہے۔

پاکستان میں ایک ہزار افراد میں سے آٹھ (8) افراد اس مرض کا شکار ہوتے ہیں۔ مردوں کے مقابلے میں عورتوں میں یہ بیماری زیادہ پائی جاتی ہے۔ سال 2011 میں لیاقت نیشنل ہسپتال میں تحقیقات کے نتائج ظاہر کرتے ہیں کہ چار ہزار نوسو (4900) افراد ڈھائ سال میں ہسپتال آئے جن میں سے (633) افراد اس بیماری کا شکارتھے۔ اس سے پتہ چلتا ہے کہ اس مرض کی شرح 12.9 فیصد ہے۔

دنیائے طب میں ہونے والی تبدیلی اور ترقی کے باوجود اس مرض کی اصل وجوہات ابھی تک سمجھ میں نہیں آئیں البتہ ہم یہ جانتے ہیں کہ ہمارے جسم کا معدافیاتی نظام اس بیماری کی وجہ ہے۔ لیکن یہ بات ابھی تک سمجھ نہیں آئ کہ یہ نظام جسم پر کس طرح حملہ آرو ہوجاتا ہے۔

تحقیقات سے یہ بات پتہ چلتی ہے کہ کچھ وجوہات اس بیماری میں ایک اہم کردار ادا کرتی ہیں اور زندگی میں ان کی موجودگی بیماری ہونے کے امکانات میں اضافہ کر دیتی ہے۔ ان میں ایک اہم وجہ خاندان خصوصاً والدین میں اس بیماری کی موجودگی ہے۔ دیگر وجوہات میں موٹاپا، عورت ہونا اور جوڑوں کا کسی چوٹ سے متاثر ہونا شامل ہے۔ تحقیقات یہ بات بھی ثابت کرتی ہیں کہ سگریٹ نوشی اس بیماری کی وجوہات میں شامل ہے۔

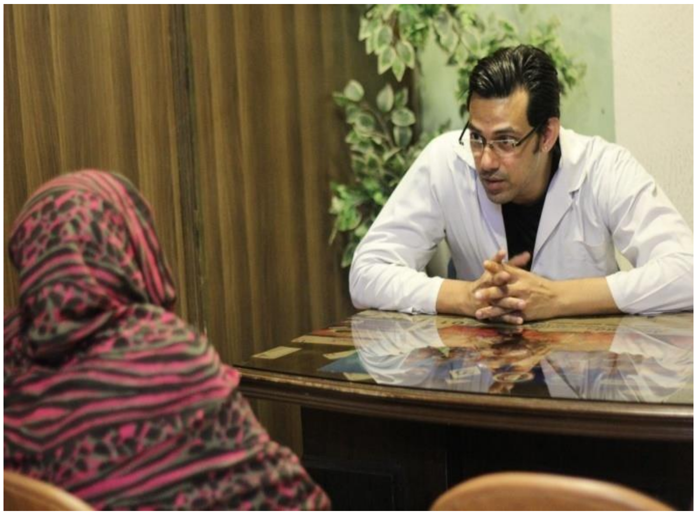

جب کوئی مریض جوڑوں کے درد کی علامات کے ساتھ ڈاکٹر کے پاس جاتا ہے تو وہ مرض کے حوالےسے اور مریض کے بارے میں کچھ سوالات پوچھتا ہے۔ ڈاکٹر متاثرہ جوڑ کی جانچ کرتا ہے تاکہ ورم کی نشاندہی ہوسکے۔

کیونکہ روماٹائڈ آرتھرائٹس کی علامات آسٹیوآرتھرائٹس اور دیگر جوڑوں کی بیماریوں سے ملتی جلتی ہیں لہٰذا مریض کی معلومات (پیشنٹ ہسٹری) اور ڈاکٹری جانچ اس بیماری کی موجودگی کو ثابت کرنے کے لئے ناکافی ہوتی ہیں لہٰذا ڈاکٹر کچھ ایکسرے اور لیباٹری ٹیسٹ کا مشورہ دیتا ہے۔

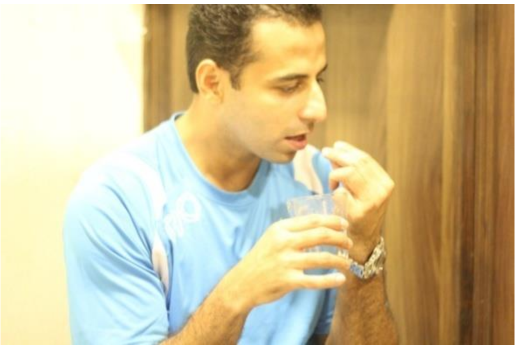

ایکسرے اور ایم-آر-آئی ان ٹیسٹ میں شامل ہے جن کا ڈاکٹر مشورہ دیتا ہے۔ یہ جوڑ کی درمیانی تنگی کو دکھاتے ہیں۔ ای-ایس-آر اور سی ریاکٹیو پروٹین لیباٹری ٹیسٹوں میں شامل ہوتے ہیں۔ روماٹائڈ فیکٹر (آر-ایف) کو خون میں معلوم کیا جاتا ہے جو بیماری کی موجودگی کو ثابت کرتا۔ یہ ٹیسٹ، مریض کی ہسٹری، جسمانی جانچ اور خاندان میں بیماری کی موجودگی مرض کو ثابت کرتے ہیں۔

بیماری کا علاج دو مقاصد کے لئے ہے:

۱۔ فوری علاج جس کا مقصد جوڑوں کا درد، ان کی سختی اور ان کے ورم میں کمی ہے۔

۲۔ دیرپہ علاج جس کا مقصد جوڑوں کو نقصان سے بچانا، ان کی حرکت میں اضافہ اور زندگی کے معیار میں بہتری ہے۔

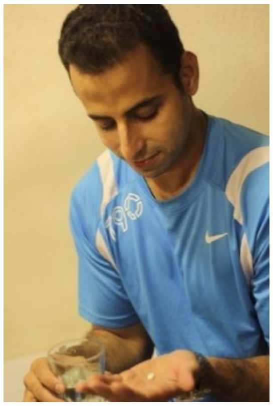

وہ ادویات جو اس مرض کے لئے استعمال کی جاتی ہیں دو اقسام کی ہوتی ہیں:

۱۔ پہلی قسم میں شامل ادویات این ایسیڈز (این-ایس-ایڈز) کہلاتی ہیں جن کا مقصد درد میں کمی ہے۔ اس بیماری میں ان ادویات کا استعمال عام ہے۔ ان کا عام مضر اثر (سائڈ آفیکٹ) معدے کا خراب ہونا اور تیزابیت ہے لہٰذا فارمسسٹ ان ادویات کو بھرے پیٹ یا کھانے کے ساتھ لینے کی تلقین کرتا ہے۔ اگر اس کے باوجود مضر اثر (سائڈ آفیکٹ) موجود رہتے ہیں دوسری قسم کی این ایسیڈز جنہیں منتخب (سلیکٹو) این ایسیڈز کہا جاتا ہے تجویز کرتا ہے۔ جن کا معدے پر مضر اثر (سائڈ آفیکٹ) نہیں ہوتا۔ اس کے علاوہ اگر مریض منہ سے دوا لینے کے حق میں نہ ہو تو درد دور کرنے والی کریم اور پٹّیاں مارکٹ میں موجود ہیں۔

۲۔ وہ دوائیں جو مرض کی شدّت میں کمی اور مرض کے بڑھنے کو روکتی ہیں مختلف اقسام کی ہوتی ہیں جن میں سے ایک قسم کورٹکواِسٹرائیڈ (کور۔ ٹی۔ کو۔ اسٹرا۔ ائیڈ) کہلاتی ہیں۔ یہ بہت طاقتور دوائیں ہیں جنہیں کم خوراک میں مختصر وقفے کے ساتھ ورم اور درد میں کمی کے لئے دیا جاتا ہے دیگر قسم کی ادویات جنہیں لمبے عرصے کے لئے دیا جاتا ہے وہ ڈیمارڈز (ڈی۔ مار۔ ڈز) کہلاتی ہیں۔

وہ افراد جن میں اس بیماری کے پیدا ہونے کے امکانات ہیں یا ایسے مریض جن میں یہ بیماری موجود ہے انہیں صحت مند اور اینٹی آکسیڈینٹ (این۔ ٹی۔ آکس۔ سی۔ ڈینٹ) سے بھرپُور غذا دی جاتی ہے۔ یہ اینٹی آکسیڈینٹ جوڑوں کے درد اور ورم میں کمی کرنے میں کردار ادا کرتے ہیں اور ان عوامل کو بھی روکتے ہیں جو اس چیز کا باعث بنتے ہیں۔ اس قسم کی غذائیں سبزیوں، پھلوں اور مچھلی پر مشتمل ہوتی ہے۔ دیگر حفاظتی طریقوں میں روزمرہ کی سرگرمیوں کے ساتھ ساتھ آرام کرنا شامل ہے۔ عام طور پر کہا جاتا ہے کہ کسی کام کے بعد دس (10) منٹ کا آرام دہ وقفہ دن بھر کی دیگر مصروفیات کو باآسانی انجام دینے میں مدد کرتا ہے۔

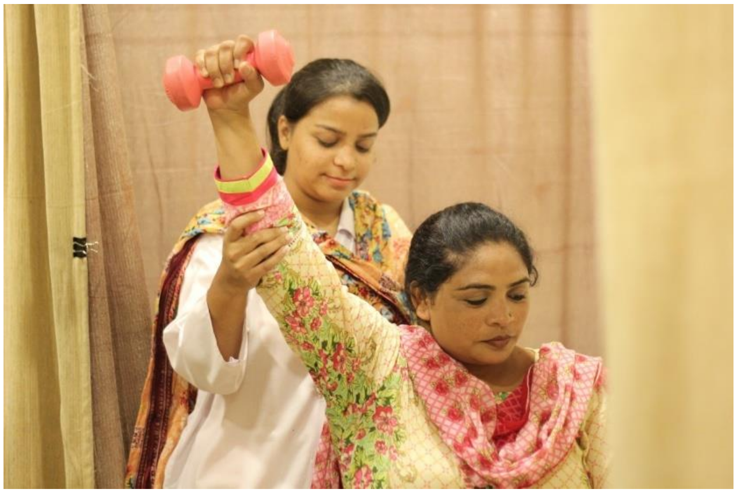

روماٹائڈآرتھرائٹس جوڑوں کی حرکت میں کمی کردیتا ہے۔ جس کی وجہ سے عضلاتی کمزوری ہونے لگتی ہے جس سے مریض کے لئے متاثرہ جوڑوں کے ساتھ دن بھر کی سرگرمیاں جاری رکھنا مشکل ہوجاتا ہے۔ ایک فزیوتھراپسٹ کی مدد سے اس سے بچاؤ ممکن ہے۔ جس کے مشورے پہ عمل کرنے سے آپ متاثرہ جوڑ اور اس سے منسلک عضلات کی ہلکی ورزش کرتے ہیں۔

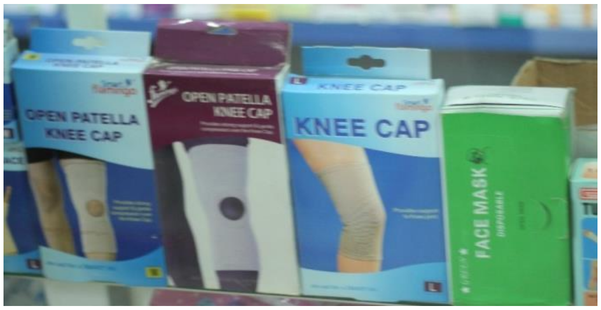

گرم اور سرد پٹّی درد اور ورم دونوں میں کمی کرتی ہے۔ گرم پٹّی جوڑ کی سختی میں کمی کرتی ہے جبکہ سرد پٹّی درد کم کرتی ہے۔ اس کے علاوہ غذا میں اضافی غضائی اجزا یا ڈائٹری سپلیمینٹس کا استعمال نرم ہڈی کے نقصان کو کم

کر کے جوڑوں کی حرکت میں اضافہ اور اس کی سختی میں کمی کردیتا ہے۔ ایک چیز کو ذہن میں رکھنا ضروری ہے کہ ایسی چیزوں کے استعمال سے پہلے ڈاکٹر سے بات کرلی جائے تاکہ وہ یہ اندازہ لگا سکے کے دیگر ادویات ان چیزوں سے متاثر تو نہیں ہورہی ہیں۔ روماٹائڈآرتھرائٹس میں دی جانے والی ادویات کا مسلسل اور صحیح استعمال علاج کے مقاصد کے حصول میں بڑی اہمیت رکھتا ہے۔

تحقیق یہ بات ثابت کرتی ہے کہ اس بیماری میں ادویات کا غلط اورغیرمُتواتِراستعمال بیماری میں اضافے اور نرم ہڈی کے نقصان کی وجہ بنتا ہے جو جوڑوں کی حرکت میں کمی کردیتا ہے اور ان کی ٹوٹ پھوٹ کا باعث بنتا ہے۔ لہٰذا یہ اہم ہے کہ آپ ادویات کی مسلسل پابندی کریں جس کی وجہ سے نہ صرف بیماری کے بڑھنے میں کمی واقعہ ہوتی ہے بلکہ یہ آپکو نارمل زندگی گزارنے میں مدد دیتی ہے۔ مریض ادویات کو دورانِ سفر اور دیگر مصروفیات میں لینا بھول جاتے ہیں۔ عید، رمضان اور دیگر تہواروں میں بھی ان ادویات کی پابندی متاثر ہوتی ہے۔ بعض مریض ان ادویات کو جان بوجھ کر چھوڑ دیتے ہیں۔ شاید اس کی وجہ مریض کا بہتری محسوس کرنا ہے۔ لہٰذا وہ یہ سوچھنے لگتا ہے کہ اب وہ ان ادویات کے بغیر بہتر رہ سکتا ہے چُنانچہ اب ان ادویات کی ضرورت نہیں ہے۔ اس کے علاوہ بعض مریض ادویات کے مضر اثرات (سائڈ اَفیکٹس) محسوس کرتے ہیں لہٰذا وہ ان ادویات کو چھوڑ دیتے ہیں کیونکہ وہ یہ سمجھتے ہیں کہ یہ ادویات غلط دی گئی ہیں۔ دراصل یہ دونوں سوچ غلط ہیں۔ آپ کو یہ بات سمجھ لینی چاہیئے کہ ان ادویات سے مسلسل جڑُے رہنے کی وجہ سے فائدہ بھی آپ کو ہوگا اوران ادویات کو چھوڑ دینے سے نقصان بھی آپ ہی اُٹھائیں گے۔

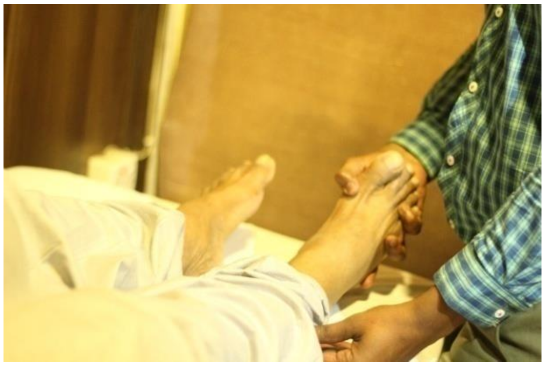

فزیوتھراپی جوڑوں اور عضلات کے لئے قوت بخش ہوتی ہے۔ روماٹائڈ آرتھرائٹس درد اور عضلات کی سختی کا سبب بنتا ہے جس کی وجہ سے مریض باآسانی حرکت نہیں کر پاتا لہٰذا اس کے عضلات (استعمال نہ ہونے کی وجہ سے) کمزور ہوجاتے ہیں جس سے مریض کے لئے روزمرہ کے کام سرانجام دینا بھی مشکل ہوجاتا ہے۔ محققین اس بات پر رضامند ہیں کہ فزیوتھراپی (ان جسمانی حرکات کی وجہ سے) جوڑوں کے درد کا باعث بنتی ہے۔ لیکن یہ بھی حقیقت ہے کہ ایسے مریض جو فزیوتھراپی کرواتے ہیں جسمانی طور سے زیادہ سرگرم ہوتے ہیں۔ ان میں درد کی برداشت اور کام کرنے کی طاقت اُن مریضوں کے مقابلے میں جو

فزیوتھراپی نہیں کرواتے زیادہ ہوتی ہے۔ لہٰذا اس کے اثرات معیارِ زندگی پر بھی ہوتے ہیں اور فزیوتھراپی کروانے والے مریض بہتر معیارِ زندگی کے ساتھ زیادہ عرصے زندہ رہتے ہیں۔ جبکہ جسمانی طور پر کمزور مریض اس سے محرُوم رہتے ہیں۔

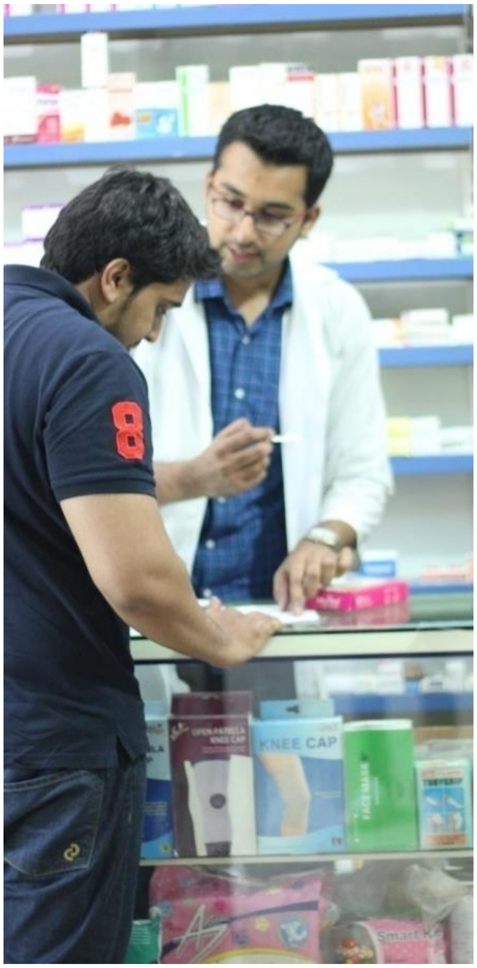

فارمسسٹ کون ہوتا ہے؟

آپ نے فارمسسٹ کی اِصطِلاح سنی ہوگی۔ لیکن کیا آپ یہ بھی جانتے ہیں کہ فارمسسٹ کون ہوتا ہے؟ کیا یہ وہ افراد ہیں جو دوائوں کی دُکان پر کھڑے ہوتے ہیں؟ ایک فارمسسٹ کیا کام کرتا ہے؟ فارمسسٹ کی ہسپتال میں ضرورت کیوں ہوتی ہے؟ ایک فارمسسٹ کا آپ کی بیماری سے کیا تعلق ہے؟ یہ وہ چند سوالات ہیں جو کسی مریض کے ذہن میں آسکتے ہیں۔ سب سے پہلے اس بات کو ذہن میں رکھئیے کہ فارمسسٹ دوائوں کی دُکان والے نہیں ہوتے۔ ان کا تعلیمی معیار ڈاکٹروں کے مساوی ہوتا ہے۔ ان کی ذمہ داریوں میں دوائیں تیار کرنا اور مہیا کرنا شامل ہے۔

اب اکثر لوگ یہ سوچھتے ہیں کہ فارمسسٹ اور دوائوں کی دُکان والے میں کیا فرق ہوتا ہے؟ حقیقت میں دونوں ایک جیسی ہی ذمہ داری اُٹھاتے ہیں لیکن مندرجہ ذیل فرق موجود ہوتا ہے:

۱۔ دوا کی دُکان والا دوا دینے کی صلاحیت رکھتا ہے لیکن فارمسسٹ نہ صرف دوا د دینے کی صلاحیت رکھتا ہے بلکہ مریض کو اُس کی بیماری کے بارے میں آکہی بھی فراہم کرتا ھے۔ فارمسسٹ مریض کو اُس کی بیماری سے متعلق مختلف باتوں پر توجہ دلاتا ہے جن کا گھر میں بخوبی خیال رکھا جاسکتا ہے۔ فارمسسٹ غذا سے تعلق رکھنے والے اور روزمرہ کی زندگی کی باتوں کی طرف توجہ مرکوُز کرواتا ہے۔ جس سے بیماری سے متعلق بعض تکالیف کو کم کرنے میں مدد ملتی ہے۔

۲۔ بعض اوقات ڈاکٹر اپنی مصروفیات کی وجہ سے مریض کو مناسب وقت نہیں دے پاتا اور مریض کی ہسٹری کے مطابق ادویات تجویز کردیتا ہے۔ ایسا کرنے سے بعض باتیں جنہیں سمجھنا ضروری ہوتا

ہے مریض سمجھ نہیں پاتا۔ ایسی صورت میں مریض کو پریشان نہیں ہونا چاہئیے بلکہ یہ سب کچھ فارمسسٹ سے پوچھنا چاہئیے جو نہ صرف دی گئی ادویات میں رہنمائی اور وضاحت کرتا ہے بلکہ علاج کے سلسلے میں دیگر معلومات دینے میں بھی ڈاکٹر کا بہترین بدل تصور کیا جاتا ہے۔

۳۔ اگر آپ کو ایسی دوائیں دے دی جائیں جو بازار میں نہ مل سکیں تو ایک فارمسسٹ ان کی جگہ دوسری ادویات تجویز کرسکتا ہے۔ ایسی صورت میں آپ کا علاج جاری رہتا ہے۔

۴۔ فارمسسٹ آپ کو دوائیں لینے کا طریقہ بھی بتاتا ہے مثلاً کون سی ادویات صبح اور کون سی رات کو لی جائیں۔ اس طرح سے دوا کا اثر بہتر ہوتا ہے۔

۵۔ ایک فارمسسٹ بعض مہنگی ادویات کو سَستی ادویات سے تبدیل کرسکتا ہے اور اس طرح سے پیسے کی بچت کرنے میں مدد کرتا ہے۔ مثال کے طور پر اگر آپ کو دی جانے والی دویا مہنگی ہے تو فارمسسٹ کسی دوسری کمپنی کی بنی ہوئی ایسی دوا تجویز کرسکتا ہے جس کے اثرات وہی ہوتے ہیں مگر قیمت کم ہوتی ہے۔

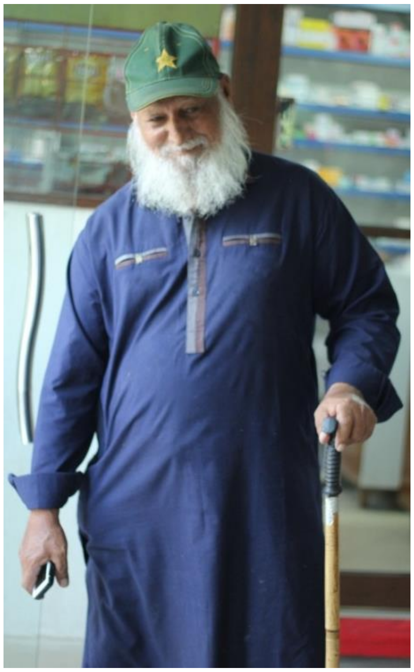

۶۔ کچھ مریض تین سے چار ادویات استعمال کرتے ہیں۔ بعض اوقات ان دوائوں کا بیک وقت استعمال جسم میں مضر اثرات (سائڈ افیکٹس) پیدا کرتے ہیں۔ مثلاً مریض پیٹ کا درد محسوس کرسکتا ہے۔ ایک فارمسسٹ ایسی صورت میں مفید مشورہ دے سکتا ہے تاکہ ان مضراثرات سے بچا جاسکے۔

۷۔ بعض ادویات غذا کے ساتھ حساس تعلق رکھتی ہیں۔ مثلاً کچھ دوائیں دودھ کے ساتھ نہیں دی جاسکتیں اور کچھ دوائیں خالی پیٹ لینا خطرناک ہوتا ہے۔ ایک فارمسسٹ ایسی ادویات کے بارے میں مفید معلومات مہیا کرسکتا ہے۔

پاکستان میں فارمسسٹ اور فارماسیوٹیکل کیرایک نیا تصور ہے۔ مریض ابھی اسکی اہمیت سے واقف نہیں ہیں۔ اب یہ وقت کا تقاضہ ہے کہ ہم فارمسسٹ پر بھروسہ کریں اور فارمسسٹ کی دی ہوئی معلومات سے استفادہ حاصل کریں لہذا اب آپ جب بھی فارمیسی جائیں تو ایک فارمسسٹ سے دوائوں کے متعلق آگہی ضرور حاصل کریں۔
