# Supplementary material for: Impact of pharmacist educational intervention on disease knowledge, rehabilitation and medication adherence, treatment-induced direct cost, health-related quality of life and satisfaction in patients with rheumatoid arthritis: study protocol for a randomized controlled trial
Source: Trials. 2019 Aug 9;20:488. doi: 10.1186/s13063-019-3540-z (PMC6688212; doi:10.1186/s13063-019-3540-z)
Supplement: Supplementary file 7 — Checklist for pharmacist intervention (to be completed by the rheumatologist). (DOCX 17 kb) [file 13063_2019_3540_MOESM7_ESM.docx]

**Checklist for pharmacist intervention (to be completed by rheumatologist)***

**Instruction for completing this checklist**

**Please read the following instruction before filling the checklist:**

1. During the intervention you are required to be silent and do not intervene.
2. You would start to fill this checklist at the time of execution of intervention by pharmacist.

**I can confirm that the pharmacist provided the following to my patient:**

- greeted the patient and made him comfortable
- asked the patient about his/her present complains
- provided a brief educational session about rheumatoid arthritis disease to patient
- explained the importance of adhering to medications prescribed
- explained the importance to physical therapy adherence
- reviewed medications explained the dosage regimen and educated patients regarding dosage schedule
- suggested generic alternatives to reduce the cost of treatment (if applicable)*
- suggested home-based self-care techniques and approaches aimed at improving productivity and mobility

**I can further confirm that:**

- the whole session was conducted in Urdu language
- the patient was able to understand the instructions given by pharmacist
- patient was able to follow the lecture pace
- the pharmacist exhibited professionalism while executing intervention
- disease education booklet was provided to patients

Sign ___________________________

Date ___________________________
